# [2.2]Paracyclophane Derivatives as Building Blocks for Coordination Polymers

**DOI:** 10.3390/ma16114051

**Published:** 2023-05-29

**Authors:** Mihail Lucian Birsa, Henning Hopf, Peter G. Jones, Laura Gabriela Sarbu, Lucian Gabriel Bahrin

**Affiliations:** 1 Department of Chemistry, Alexandru Ioan Cuza University of Iasi, No. 11 Carol I Blvd., 700506 Iasi, Romania; 2 Institute of Organic Chemistry, Technical University of Braunschweig, Hagenring 30, D-38106 Braunschweig, Germany; 3 Institute of Inorganic and Analytical Chemistry, Technical University of Braunschweig, Hagenring 30, D-38106 Braunschweig, Germany; 4 Center of Advanced Research in Bionanoconjugates and Biopolymers—Intelcenter, Petru Poni Institute of Macromolecular Chemistry, No. 41A Grigore Ghica Voda Alley, 700487 Iasi, Romania

**Keywords:** [2.2]paracyclophane, coordination polymer, Suzuki coupling

## Abstract

Several new di- and tetracarboxylic [2.2]paracyclophane derivatives were obtained via Suzuki coupling between the appropriately brominated [2.2]paracyclophanes and 4-(methoxycarbonyl)phenylboronic acid. The reaction of one of these products, namely *pp*-bis(4-carboxyphenyl)[2.2]paracylophane (**12**), with zinc nitrate afforded a 2D coordination polymer comprising zinc-carboxylate paddlewheel clusters linked together by cyclophane cores. The zinc center is five-coordinated in a square-pyramidal geometry, with a DMF oxygen atom at the apex and four carboxylate oxygen atoms in the base.

## 1. Introduction

First discovered in 1949 by Brown and Farthing [1], [2.2]paracyclophane consists of two benzene rings bound to each other by two ethylene bridges at positions 1 and 4. The close proximity of the two aromatic rings makes the [2.2]paracyclophane core a strained molecule, with a strain energy of 31 kcal/mol [2]. Each of the two benzene rings is bent out of plane to a flattened boat conformation, whereby the distance between them varies from 2.78 Å to 3.09 Å, which is smaller than the expected 3.40 Å, representing the sum of the van der Waals radii for carbon atoms [3]. The two ethylene bridges prevent the free rotation of the aromatic rings, and the compound is thus stable up to approximately 200 °C. However, above 180–200 °C, pyrolysis of one of the bridges can occur, allowing the aromatic rings to rotate freely. This phenomenon is used in the synthesis of various substituted [2.2]paracyclophane derivatives by allowing one ethylene bridge to break and then re-form upon cooling [4]. Some substituted [2.2]paracyclophanes (e.g., monosubstituted systems) display planar chirality, which makes them attractive in the design of chiral ligands for asymmetric synthesis [5]. Other areas in which [2.2]paracyclophane derivatives have found use are polymer chemistry [6], as circularly polarized luminescence fluorophores [7], optoelectronics [8] and as coenzyme analogues [9].

Although widely used as ligands in catalytic systems, [2.2]paracyclophane derivatives have seen only limited use in metal–organic framework (MOF) design, with the few exceptions focusing mainly on the metals zirconium [10,11], silver [12,13,14,15], cobalt [16] and copper [17].

In the past few years, our group has taken an interest in the field of MOFs, particularly in investigating new structures based on mesitylene-derived linkers [18,19,20,21,22]. Combining this with our ongoing interest in the field of [2.2]paracyclophanes [23,24,25], we decided to synthesize and characterize several di- and tetracarboxylic [2.2]paracyclophanes and assess their potential in MOF synthesis.

## 2. Materials and Methods

### 2.1. Chemistry

[2.2]Paracyclophane (lot no. 4348995, Karlsruhe, Germany) was purchased from Alfa Aesar. 4-(Methoxycarbonyl)-phenylboronic acid was purchased from TCI (lot no. JB3LB-LE, Shanghai, China). All other reagents were purchased from Sigma Aldrich (St. Louis, MO, USA).

Melting points were obtained on a *KSPI* melting-point meter (A.KRÜSS Optronic, Hamburg, Germany) and are uncorrected. IR spectra were recorded on a Bruker Tensor 27 instrument (Bruker Optik GmbH, Ettlingen, Germany). The NMR spectra were recorded on a Bruker NEO 400 instrument (Bruker BioSpin, Rheinstetten, Germany) operating at 400.1 and 100.6 MHz for ^1^H and ^13^C nuclei, respectively. Chemical shifts are reported in ppm downfield from TMS. Mass spectra were recorded on a Thermo Scientific ISQ LT instrument (Thermo Fisher Scientific Inc., Waltham, MA, USA).

NMR spectra for compounds **5**–**12**, **14**, **15**, **17** and **18** (Figures S1–S24), as well as IR spectra for compounds **5**–**12**, **14**, **15**, **17, 18** and **19** (Figures S25–S37) can be found in the Supplementary Materials.

#### 2.1.1. General Procedure A—Suzuki Coupling; Compound **8**

A 20 mL Schlenck tube containing *pp*-dibromo[2.2]paracyclophane (183 mg, 0.5 mmol), 4-(methoxycarbonyl)phenylboronic acid (270 mg, 1.5 mmol), sodium carbonate (318 mg, 3 mmol, anhydrous) and SPhosPdG4 (20 mg) was placed under high vacuum and backfilled with nitrogen. After 3 cycles, DMF (10 mL, anhydrous) was added under nitrogen to the flask via a syringe, and the reaction mixture was heated to 120 °C with stirring for 24 h. The contents of the flask were then cooled down to room temperature and precipitated in water (200 mL, under stirring). The precipitate thus formed was filtered off, dried in air and subjected to flash chromatography using a mixture of dichloromethane/hexane (80/20, *v*/*v*) as a mobile phase. The desired material was isolated as a mixture alongside the homocoupling product. Recrystallization of this mixture from ethyl acetate yielded **8** in the form of yellow crystals (35%). M.p. 292–293 °C. ^1^H NMR (400 MHz, CDCl_3_): *δ* 8.23–8.16 (4H, m, H-ar), 7.66–7.59 (4H, m, H-ar), 6.75–6.69 (4H, m, H-ar), 6.61 (2H, dd, ^3^*J* = 7.82 Hz, ^4^*J* = 1.61 Hz, H-ar), 4.00 (6H, s, 2CH_3_), 3.52–3.38 (2H, m, CH_2_), 3.15–3.03 (2H, m, CH_2_), 2.97–2.84 (2H, m, CH_2_), 2.84–2.72 (2H, m, CH_2_) ppm. ^13^C NMR (100 MHz, CDCl_3_): *δ* 167.2, 145.8, 141.1, 140.0, 137.1, 135.0, 132.3, 129.9, 129.8, 129.7, 128.6, 52.2, 34.7, 33.8 ppm. IR-ATR (cm^−1^): ῦ = 2950 (aliphatic C-H stretching), 2159, 1714 (C=O stretching), 1605 (C=C stretching), 1432 (aliphatic C-H bending), 1274 (C-O stretching), 1100 (C-O stretching), 857 (aromatic C-H bending), 711 (aromatic C-H bending), 484. EI-MS *m/z* (%): 476 (M^+^, 1), 444 (28), 237 (7), 206 (42), 178 (100).

#### 2.1.2. Compound **5**

General procedure A. White solid (12%). M.p. 225–226 °C. ^1^H NMR (400 MHz, CDCl_3_): *δ* 7.83–7.78 (4H, m, H-ar), 7.37–7.32 (4H, m, H-ar), 6.82 (2H, d, ^3^*J* = 7.60 Hz, H-ar), 6.81 (2H, d, ^4^*J* = 1.73 Hz, H-ar), 6.72 (2H, dd, ^3^*J* = 7.60 Hz, ^4^*J* = 1.73 Hz, H-ar), 3.93 (6H, s, 2CH_3_), 3.34–3.27 (2H, m, CH_2_), 3.27–3.21 (2H, m, CH_2_), 3.20–3.10 (4H, m, 2CH_2_) ppm. ^13^C NMR (100 MHz, CDCl_3_): *δ* 167.0, 144.0, 139.6, 139.5, 137.2, 136.2, 132.9, 130.4, 129.3, 129.1, 128.1, 52.0, 35.2, 35.0 ppm. IR-ATR (cm^−1^): ῦ = 2971 (aliphatic C-H stretching), 1713 (C=O stretching), 1607 (C=C stretching), 1434 (aliphatic C-H bending), 1273 (C-O stretching), 1094 (C-O stretching), 858 (aromatic C-H bending), 771 (aromatic C-H bending), 705 (aromatic C-H bending). EI-MS *m/z* (%): 476 (M^+^, 3), 444 (25), 237 (49), 206 (18), 178 (100).

#### 2.1.3. Compound **6**

General procedure A. White solid (30%). M.p. 199–200 °C. ^1^H NMR (400 MHz, CDCl_3_): *δ* 8.12–8.04 (4H, m, H-ar), 7.45–7.37 (4H, m, H-ar), 6.78 (2H, d, ^3^*J* = 7.69 Hz, H-ar), 6.71 (2H, dd, ^3^*J* = 7.70 Hz, ^4^*J* = 1.41 Hz, H-ar), 6.67 (2H, d, ^4^*J* = 1.40 Hz, H-ar), 3.98 (6H, s, 2CH_3_), 3.60–3.50 (2H, m, CH_2_), 3.22–3.11 (2H, m, CH_2_), 3.09–2.97 (2H, m, CH_2_), 2.83–2.72 (2H, m, CH_2_) ppm. ^13^C NMR (100 MHz, CDCl_3_): *δ* 167.1, 145.6, 139.7, 139.4, 137.2, 135.8, 133.0, 130.0, 129.8, 129.0, 128.4, 52.2, 34.5, 34.3 ppm. IR-ATR (cm^−1^): ῦ = 2947 (aliphatic C-H stretching), 1712 (C=O stretching), 1607 (C=C stretching), 1433 (aliphatic C-H bending), 1272 (C-O stretching), 1105 (C-O stretching), 1017 (C-O stretching), 856 (aromatic C-H bending), 775 (aromatic C-H bending), 693, 456. EI-MS *m/z* (%): 476 (M^+^, 2), 444 (32), 237 (8), 206 (41), 178 (100).

#### 2.1.4. Compound **7**

General procedure A. White solid (36%). M.p. 272–273 °C. ^1^H NMR (400 MHz, CDCl_3_): *δ* 8.20–8.13 (4H, m, H-ar), 7.62–7.55 (4H, m, H-ar), 6.77 (2H, d, ^4^*J* = 1.83 Hz, H-ar), 6.70 (2H, d, ^3^*J* = 7.82 Hz, H-ar), 6.66 (2H, dd, ^3^*J* = 7.79 Hz, ^4^*J* = 1.80 Hz, H-ar), 3.99 (6H, s, 2CH_3_), 3.34–3.23 (2H, m, CH_2_), 3.23–3.08 (2H, m, 2CH_2_), 2.59–2.47 (2H, m, CH_2_) ppm. ^13^C NMR (100 MHz, CDCl_3_): *δ* 167.1, 145.6, 141.5, 139.9, 137.5, 132.5, 132.0, 131.8, 129.9, 129.6, 128.6, 52.2, 35.1, 33.5 ppm. IR-ATR (cm^−1^): ῦ = 2927 (aliphatic C-H stretching), 1715 (C=O stretching), 1604 (C=C stretching), 1432 (aliphatic C-H bending), 1276 (C-O stretching), 1102 (C-O stretching), 967, 858 (aromatic C-H bending), 707 (aromatic C-H bending), 454. EI-MS *m/z* (%): 476 (M^+^, 4), 444 (25), 350 (23), 237 (28), 207 (54), 178 (100).

#### 2.1.5. Compound **14**

General procedure A (double the amount of 4-(methoxycarbonyl)-phenylboronic acid and catalyst). White solid (28%). M.p. 320–321 °C. ^1^H NMR (400 MHz, CDCl_3_): *δ* 8.00–7.82 (8H, m, H-ar), 7.46–7.08 (8H, m, H-ar), 6.81 (4H, s, H-ar), 3.92 (12H, s, 4CH_3_), 3.11–2.88 (8H, m, 4CH_2_) ppm. ^13^C NMR (100 MHz, CDCl_3_): *δ* 166.9, 144.5, 139.1, 138.4, 131.5, 128.9, 128.1, 52.1, 33.5 ppm. IR-ATR (cm^−1^): ῦ = 2954 (aliphatic C-H stretching), 1717 (C=O stretching), 1605 (C=C stretching), 1434 (aliphatic C-H bending), 1270 (C-O stretching), 1101 (C-O stretching), 1016, 958, 857 (aromatic C-H bending), 765 (aromatic C-H bending), 720 (aromatic C-H bending), 466. EI-MS *m/z* (%): 744 (M^+^, 2), 444 (16), 372 (15), 339 (60), 281 (63), 253 (100), 178 (80).

#### 2.1.6. Compound **17**

General procedure A (double the amount of 4-(methoxycarbonyl)-phenylboronic acid and catalyst). White solid (30%). M.p. 296–297 °C. ^1^H NMR (400 MHz, CDCl_3_): *δ* 8.14–8.06 (8H, m, H-ar), 7.50–7.43 (8H, m, H-ar), 6.89 (4H, s, H-ar), 4.00 (12H, s, 4CH_3_), 3.63–3.50 (4H, m, 2CH_2_), 2.91–2.76 (4H, m, 2CH_2_) ppm. ^13^C NMR (100 MHz, CDCl_3_): *δ* 166.9, 144.7, 139.6, 137.5, 132.6, 130.0, 128.8, 52.2, 33.4 ppm. IR-ATR (cm^−1^): ῦ = 2955 (aliphatic C-H stretching), 1714 (C=O stretching), 1605 (C=C stretching), 1434 (aliphatic C-H bending), 1268 (C-O stretching), 1103 (C-O stretching), 1012, 868 (aromatic C-H bending), 729 (aromatic C-H bending), 467. EI-MS *m/z* (%): 744 (M^+^, 3), 444 (5), 371 (7), 339 (32), 312 (36), 281 (42), 253 (100).

#### 2.1.7. General Procedure B—Ester Hydrolysis; Compound **12**

Ester **8** (367 mg, 0.77 mmol) was dissolved in a mixture of THF (30 mL) and methanol (10 mL). To this, a solution of sodium hydroxide (92 mg, 2.3 mmol) in water (4 mL) was added, and the resulting mixture was heated at 60 °C for 24 h. The contents of the flask were then poured into water (400 mL) with stirring, followed by the addition of hydrochloric acid (37%, 1 mL). The resulting precipitate was then stirred at room temperature for 30 min, filtered, washed thoroughly with water and air-dried to yield pure **12** as a white solid (95%). Needle-like crystals of **12** were obtained by recrystallization from DMF. M.p. 352–353 °C. ^1^H NMR (400 MHz, DMSO-*d*_6_): *δ* 12.99 (2H, bs, COOH), 8.12–8.06 (4H, m, H-ar), 7.73–7.65 (4H, m, H-ar), 6.86 (2H, d, ^3^*J* = 7.8 Hz, H-ar), 6.76 (2H, d, ^4^*J* = 1.8 Hz, H-ar), 6.50 (2H, dd, ^3^*J* = 7.8 Hz, ^4^*J* = 1.8 Hz, H-ar), 3.35–3.25 (2H, m, CH_2_), 3.14–3.03 (2H, m, CH_2_), 2.96–2.83 (2H, m, CH_2_), 2.70–2.60 (2H, m, CH_2_) ppm. ^13^C NMR (100 MHz, DMSO-*d*_6_): *δ* 167.8, 145.5, 141.2, 140.0, 135.6, 132.5, 130.2, 130.0, 129.6, 34.6, 33.7 ppm. IR-ATR (cm^−1^): ῦ = 2925 (aliphatic C-H stretching), 1674 (C=O stretching), 1605 (C=C stretching), 1423 (aliphatic C-H bending), 1282 (C-O stretching), 780 (aromatic C-H bending), 717 (aromatic C-H bending), 546. EI-MS *m/z* (%): 448 (M^+^, 1), 430 (15), 223 (13), 178 (100).

#### 2.1.8. Compound **9**

General procedure B. White solid (89%). M.p. 327–328 °C. ^1^H NMR (400 MHz, DMSO-*d*_6_): *δ* 12.79 (2H, bs, COOH), 7.75–7.67 (4H, m, H-ar), 7.55–7.45 (4H, m, H-ar), 6.95 (2H, d, ^4^*J* = 1.75 Hz, H-ar), 6.87 (2H, d, ^3^*J* = 7.57 Hz, H-ar), 6.72 (2H, dd, ^3^*J* = 7.58 Hz, ^4^*J* = 1.72 Hz, H-ar), 3.19–3.05 (8H, m, 4CH_2_) ppm. ^13^C NMR (100 MHz, DMSO-*d*_6_): *δ* 167.6, 143.6, 139.5, 139.5, 137.3, 136.7, 133.2, 130.3, 129.7, 129.3, 129.0, 34.9 ppm. IR-ATR (cm^−1^): ῦ = 2981 (aliphatic C-H stretching), 1682 (C=O stretching), 1608 (C=C stretching), 1425 (aliphatic C-H bending), 1299 (C-O stretching), 759 (aromatic C-H bending), 727 (aromatic C-H bending), 552. EI-MS *m/z* (%): 448 (M^+^, 3), 430 (18), 223 (24), 207 (14), 178 (100).

#### 2.1.9. Compound **10**

General procedure B. White solid (99%). M.p. 355–356 °C. ^1^H NMR (400 MHz, DMSO-*d*_6_): *δ* 13.24–12.74 (2H, bs, COOH), 8.04–7.94 (4H, m, H-ar), 7.47–7.35 (4H, m, H-ar), 6.81 (2H, d, ^3^*J* = 7.63 Hz, H-ar), 6.74 (2H, dd, ^3^*J* = 7.66 Hz, ^4^*J* = 1.77 Hz, H-ar), 6.60 (2H, d, ^4^*J* = 1.78 Hz, H-ar), 3.49–3.37 (2H, m, CH_2_), 3.15–2.97 (4H, m, 2CH_2_), 2.77–2.65 (2H, m, CH_2_) ppm. ^13^C NMR (100 MHz, DMSO-*d*_6_): *δ* 167.7, 145.2, 139.9, 139.2, 137.4, 136.3, 133.5, 130.1, 130.0, 129.4, 129.3, 34.3, 34.1 ppm. IR-ATR (cm^−1^): ῦ = 2924 (aliphatic C-H stretching), 1683 (C=O stretching), 1606 (C=C stretching), 1418 (aliphatic C-H bending), 1281 (C-O stretching), 855 (aromatic C-H bending), 777 (aromatic C-H bending), 720 (aromatic C-H bending), 502. EI-MS *m/z* (%): 448 (M^+^, 3), 430 (35), 206 (32), 178 (100).

#### 2.1.10. Compound **11**

General procedure B. White solid (82%). M.p. 343–344 °C. ^1^H NMR (400 MHz, DMSO-*d*_6_): *δ* 13.26–12.67 (2H, bs, COOH), 8.12–8.02 (4H, m, H-ar), 7.70–7.60 (4H, m, H-ar), 6.81 (2H, d, ^4^*J* = 1.85 Hz, H-ar), 6.77 (2H, dd, ^3^*J* = 7.75 Hz, ^4^*J* = 1.80 Hz, H-ar), 6.59 (2H, d, ^3^*J* = 7.70 Hz, H-ar), 3.26–3.15 (2H, m, CH_2_), 3.15–3.01 (4H, m, 2CH_2_), 2.48–2.37 (2H, m, CH_2_) ppm. ^13^C NMR (100 MHz, DMSO-*d*_6_): *δ* 167.8, 145.3, 141.4, 140.3, 137.2, 132.7, 132.4, 132.3, 130.2, 130.0, 129.6, 34.8, 33.5 ppm. IR-ATR (cm^−1^): ῦ = 2927 (aliphatic C-H stretching), 1627, 1605 (C=C stretching), 1422 (aliphatic C-H bending), 1290 (C-O stretching), 861 (aromatic C-H bending), 779 (aromatic C-H bending), 719 (aromatic C-H bending), 486. EI-MS *m/z* (%): 448 (M^+^, 2), 430 (17), 223 (23), 207 (12), 178 (100).

#### 2.1.11. Compound **15**

General procedure B (double the amount of sodium hydroxide). White solid (62%). M.p. 352–353 °C. ^1^H NMR (400 MHz, DMSO-*d*_6_): *δ* 13.60–12.10 (4H, bs, COOH), 8.08–6.49 (20H, m, H-ar), 3.12–2.98 (4H, m, 2CH_2_), 2.87–2.70 (4H, m, 2CH_2_) ppm. ^13^C NMR (100 MHz, DMSO-*d*_6_): *δ* 167.6, 144.5, 139.0, 138.4, 131.9, 129.0, 128.9, 33.3 ppm. IR-ATR (cm^−1^): ῦ = 1695 (C=O stretching), 1606 (C=C stretching), 1418 (aliphatic C-H bending), 1294 (C-O stretching), 858 (aromatic C-H bending), 755 (aromatic C-H bending), 715 (aromatic C-H bending), 469. EI-MS *m/z* (%): 688 (M^+^, 1), 475 (10), 444 (16), 178 (100).

#### 2.1.12. Compound **18**

General procedure B (double the amount of sodium hydroxide). White solid (85%). M.p. 358–359 °C. ^1^H NMR (400 MHz, DMSO-*d*_6_): *δ* 13.52–12.49 (4H, bs, COOH), 8.03–7.95 (8H, m, H-ar), 7.56–7.43 (8H, m, H-ar), 6.88 (4H, s, H-ar), 3.55–3.41 (4H, m, 2CH_2_), 2.94–2.80 (4H, m, 2CH_2_) ppm. ^13^C NMR (100 MHz, DMSO-*d*_6_): *δ* 167.6, 144.3, 139.5, 137.8, 132.8, 130.2, 129.8, 129.3, 31.2 ppm. IR-ATR (cm^−1^): ῦ = 1683 (C=O stretching), 1606 (C=C stretching), 1424 (aliphatic C-H bending), 1298 (C-O stretching), 864 (aromatic C-H bending), 781 (aromatic C-H bending), 735 (aromatic C-H bending), 421. EI-MS *m/z* (%): 688 (M^+^, 1), 509 (7), 444 (18), 372 (23), 253 (52), 207 (25), 178 (100).

#### 2.1.13. Compound **19**—2D Zinc Coordination Polymer

Linker **12** (recrystallized, 29 mg, 0.05 mmol) and zinc nitrate hexahydrate (30 mg, 0.1 mmol) were placed in a 12 mL vial and dissolved in a mixture of DMF (4 mL), ethanol (1 mL) and water (1 mL). The vial was then capped and heated to 80 °C for 48 h, during which crystals formed in the reaction vessel. The vial was then cooled to room temperature, and the content was filtered, washed with DMF and air-dried, yielding **19** as a crystalline white solid (26 mg, 69%). IR-ATR (cm^−1^): ῦ = 2966 (aliphatic C-H stretching), 1603 (C=C stretching), 1398 (aliphatic C-H bending), 788 (aromatic C-H bending), 696 (aromatic C-H bending), 473.

### 2.2. X-ray Crystallography

Crystals were mounted in inert oil on nylon loops and transferred to the cold gas stream of a Rigaku/Oxford XtaLAB Synergy diffractometer (Rigaku Oxford Diffraction, Wrocław, Poland). Absorption corrections were implemented on the basis of multi-scans. The structures were refined anisotropically on *F*^2^ using the program SHELXL-2018 [26]. Because of the known distortion of the cyclophane framework, the hydrogen atoms of the cyclophane rings were refined freely (but with C-H distances restrained to be approximately equal using the command “SADI”). Other hydrogen atoms were included using rigid methyl groups or a riding model starting from calculated positions. Crystal data and refinement details are given in Table 1 and Table 2.

#### Special Features and Exceptions

**5**: The Flack parameter, determined on the basis of the weak anomalous dispersion of the oxygen atoms, refined to −0.21(13).

**7**: The crystal was twinned by 180° rotation around the *b* axis. The structure was refined using the “HKLF 5” method, whereby the relative volume of the larger twin component refined to 0.5100(7). Because the components were approximately equal, all reflections were included in the dataset (overlapped reflections together with non-overlapped reflections from both components). Because of the usual features of the “HKLF 5” refinement, the number of reflections should be interpreted with caution, and *R*_int_ does not apply.

**12·2DMF**: The hydrogen atom at O_2_ was refined freely. A significant peak of residual electron density (1.0 e/Å^3^) was identified as a disordered position of the DMF oxygen atom. This would correspond to an alternative arrangement whereby the O and OH groups at C17 and the H and O atoms at C93 “exchange” places. A feasible disorder model was refined, leading to an occupation factor of only 0.054(2) for the minor component, the dimensions of which are, however, not very satisfactory. We therefore regard the disorder model as tentative.

**14**: The ester group at C27 is disordered over two positions with occupancies of 0.485 and 0.515(19). Appropriate restraints were employed to improve the stability of refinement, but the dimensions of disordered groups should always be interpreted with caution.

**19**: The methyl group at C17 displays a large displacement factor component perpendicular to the mirror plane in which it lies. Attempts to refine a disorder model were, however, unsatisfactory and led to higher *R* values.

Crystallographic data are summarized in Table 1 and Table 2. Additionally, complete data have been deposited with the Cambridge Crystallographic Data Centre under the numbers CCDC 2245482–2245488. Copies of the data can be obtained free of charge at www.ccdc.cam.ac.uk/data_request/cif.

## 3. Results and Discussion

**Scheme 1:**
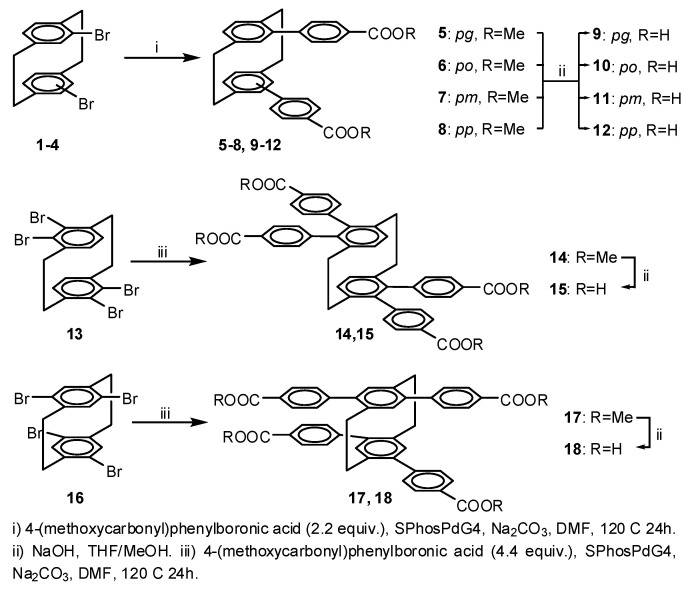
Synthesis of the di- and tetrasubstituted carboxy [2.2]paracyclophanes. *pg* (*pseudo-gem*)–4,15–disubstituted [2.2]paracyclophane; *po* (*pseudo-ortho*)–4,16–disubstituted [2.2]paracyclophane; *pm* (*pseudo-meta*)–4,13–disubstituted [2.2]paracyclophane; *pp* (*pseudo-para*)–4,12–disubstituted [2.2]paracyclophane.

Four linkers containing two carboxyl groups were obtained from the corresponding *pg*-, *po*-, *pm*- and *pp*-dibromo[2.2]paracyclophane via Suzuki coupling with 4-(methoxycarbonyl)phenylboronic acid. Moreover, two tetracarboxylic linkers were obtained using appropriately substituted tetrabromo[2.2]paracyclophane as starting material, again via Suzuki coupling with 4-(methoxycarbonyl)phenylboronic acid (Scheme 1). At the time we were working on these systems, derivatives **12**, **15** and **17** were also being investigated by other groups [10,11]. Nevertheless, we present a more in-depth characterization of these organic linkers, including the single-crystal X-ray structure of **12**.

Attempts to couple brominated paracyclophanes **7** or **8** with 4-carboxyphenylboronic acid were unsuccessful, regardless of the solvent system (dioxane/ethanol/water, DMSO/water, *i*-propanol/water), base (Na_2_CO_3_, K_2_CO_3_, K_3_PO_4_) or catalyst ([Pd(PPh_3_)_4_], XPhosPdG4, SPhosPdG4). After each trial, no desired product could be isolated from the reaction mixture. Furthermore, the starting material could not be recovered. As a consequence, we turned our attention to an indirect route to the desired carboxylic acids via the corresponding methyl esters. Reactions using 4-(methoxycarbonyl)phenylboronic acid as a coupling partner proved to be successful when using dry, degassed DMF and anhydrous sodium carbonate as a base. However, despite our best efforts, each coupling reaction we performed was accompanied by the formation of the homocoupling product of 4-(methoxycarbonyl)phenylboronic acid along with the desired paracyclophane derivative. This proved problematic during workup, as the R_f_ value of the homocoupling product was very similar to that of paracyclophanes **5**–**8**. This meant that after flash chromatography, the desired product was obtained as a mixture with 4,4′-bis(methoxycarbonyl)biphenyl. In order to isolate paracyclophane esters **5**–**8**, the mixture had to be further recrystallized from toluene or ethyl acetate, which afforded the target esters as crystals. Although the yields calculated from the ^1^H NMR spectra of the reaction mixtures were around 60% for paracyclophanes **6**–**8** (*po*, *pm* and *pp* isomers), the practical yields after recrystallization were only around 30–35%. As far as the *pg* isomer **5** is concerned, the isolated yield was only around 12%, presumably because of steric hindrance. With regard to the tetracarboxylic products **14** and **17**, we were able to isolate these from the homocoupling product using flash chromatography; however, the isolated fractions still contained some unidentified impurities, so once again, recrystallization was necessary, leading to isolated yields of around 28–30%, despite ^1^H NMR data of the crude products indicating a total yield of around 55–60%.

The ^1^H NMR characterization of products **5**–**8** revealed the expected patterns for the substituted [2.2]paracyclophane core between 6.60–6.90 ppm as two doublets and a doublet of doublets (^4^*J* around 1.4–1.8 Hz and ^3^*J* around 7.6–7.8 Hz). The protons of the newly introduced benzene rings could be observed as two multiplets around 7.30–7.60 ppm and 8.10–8.20 ppm. The three protons of the ester functionality were found around 3.90–4.00 ppm as singlets, while the protons belonging to the ethylene bridges were observed between 2.50–3.60 ppm as multiplets. The ^13^C NMR spectra of **5**–**8** revealed the carbonyl carbon atom at around 167.0 ppm, while the aromatic carbon atoms were found between 125.0 ppm and 146.0 ppm. The signals for the methoxy group carbon atom were observed around 52.0 ppm, while the bridge carbon atoms were found around 34.0–35.0 ppm.

The NMR spectra of tetracoupled products **14** and **17** revealed the signals of the [2.2]paracyclophane aromatic protons as singlets at 6.81 ppm and 6.89 ppm, respectively, while the protons belonging to the newly coupled aromatic rings were observed as two multiplets around 7.20 ppm and 7.90 ppm for **14** and 7.50 ppm and 8.10 ppm for **17**. The methoxy group protons were found at 3.92 ppm and 4.00 ppm, while the bridge protons were observed as multiplets between 2.80 ppm and 3.60 ppm. Similarly to the previously discussed derivatives, the carbonyl carbon atoms for esters **14** and **17** were found around 167.0 ppm, while the aromatic carbon atoms were observed between 128.0 ppm and 145.0 ppm. The methoxy group carbon atom was present at around 52.0 ppm, with the bridge carbon atoms at about 33.4 ppm.

The hydrolysis of esters **5**–**8, 14** and **17** yielded the desired acids **9**–**12, 15** and **18**, respectively. The ^1^H NMR spectra of the acids displayed new signals in the form of broad singlets at around 13.0 ppm, corresponding to the carboxylic protons. Moreover, the signals belonging to the ester methoxy group were no longer present. In the cases of **12, 15** and **18**, the recorded data correspond to those previously published [10,11].

The EI-MS analysis revealed that esters **5**–**8** lost a methoxy fragment, giving a signal at *m/z* = 444 with an intensity of 25–32%. The cleavage of the two ethylene bridges gave rise to a signal at *m/z* = 237, with an intensity ranging between 7 and 49%. The loss of both 4-(methoxycarbonyl)-phenyl fragments led to a signal at *m/z* = 206, with an intensity of 18–42%. The further loss of an ethylene bridge induced the appearance of the most intense signal (100%) at *m/z* = 178. For esters **14** and **17**, the cleavage of both ethylene bridges led to a signal at *m/z* = 372 with an intensity of 10–15%. The loss of three 4-(methoxycarbonyl)-phenyl fragments gave rise to a signal at *m/z* = 339, with an intensity of 32–60%. The further loss of a methoxycarbonyl fragment led to a signal at *m/z* = 281, which had an intensity of 42–63%. Finally, the most intense signal (100%) appeared at *m/z* = 253 through the further loss of an ethylene bridge. Carboxylic acids **9**–**12**, through the loss of a methoxy fragment, led to a signal at *m/z* = 430 with an intensity of 15–35%. Similarly to esters **5**–**8**, the most intense signal (100%) could be found at *m/z* = 178, which also held true for tetracarboxylic acids **15** and **18**.

### X-ray Crystallography

Upon recrystallizing esters **5**–**8** and **14** from ethyl acetate, single crystals suitable for X-ray diffraction were obtained. Furthermore, single crystals suitable for X-ray diffraction were also obtained for acid **12** by recrystallization from DMF.

The atom numbering of the [2.2]paracyclophane unit is a potential source of confusion because the “second” ring C11-C16 can be numbered in various ways. We chose the generally preferred method with bridgehead atoms C3 opposite C14 and C6 opposite C11 and the substituent of the first ring at C4. Atoms C12, C13, C15 and C16 are then assigned such that the atom numbers increase in the same sense as the first ring when the molecule is viewed perpendicular to the rings. For molecules with crystallographic symmetry, however, this does not apply because the second ring needs no numbering.

All structures display the usual features corresponding to the strain of [2.2]-paracyclophane ring systems, e.g., lengthened single bonds and increased angles in the bridges, flattened boat conformations for the rings and decreased ring angles at the bridgehead atoms.

**Figure 1:**
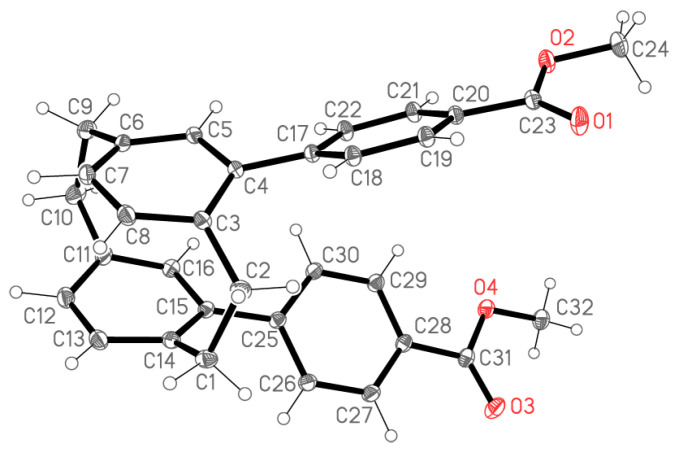
Structure of compound **5** in the crystal. In all figures, ellipsoids represent 50% probability levels.

**Figure 2:**
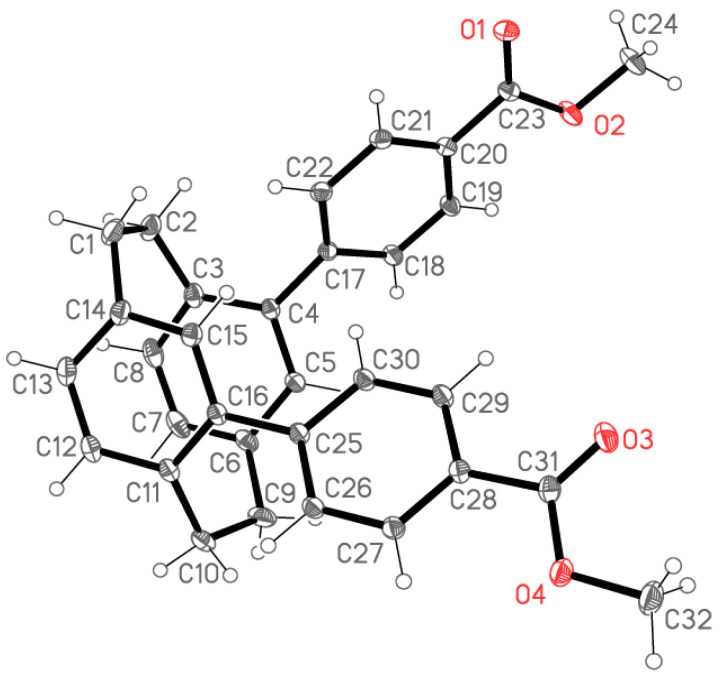
Structure of compound **6** in the crystal.

**Figure 3:**
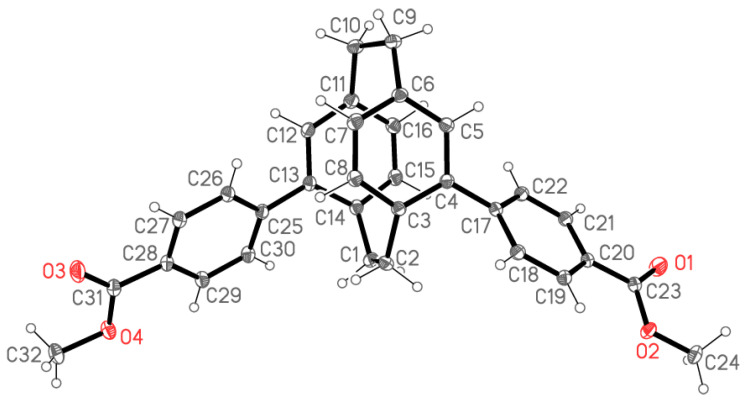
Structure of compound **7** in the crystal.

**Figure 4:**
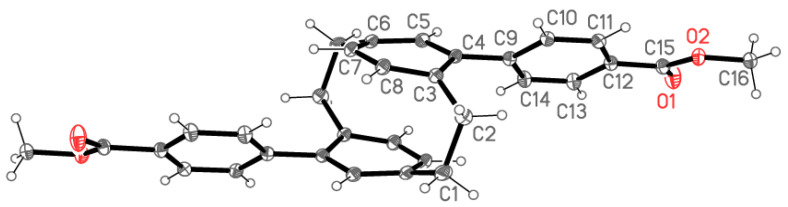
Structure of compound **8** in the crystal. Only the asymmetric unit is numbered.

Compounds **5**–**8** (Figure 1, Figure 2, Figure 3 and Figure 4) represent all four possible isomers of a [2.2]-paracyclophane system with two identical substituents, one on each ring. The substitution patterns can be described as pseudo-*geminal* (4,15), pseudo-*ortho* (4,16), pseudo-*meta* (4,13) and pseudo-*para* (4,12), respectively, based on the relative positions of the substituents when the molecule is viewed perpendicular to the rings. Compound **8** possesses crystallographic inversion symmetry; compound **5** approximate mirror symmetry; and compound **7** approximate twofold symmetry.

**Figure 5:**
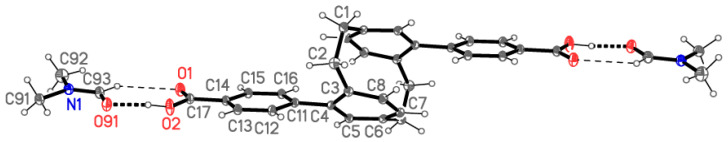
Structure of compound **12·2DMF** in the crystal. Only the asymmetric unit is numbered.

**Figure 6:**
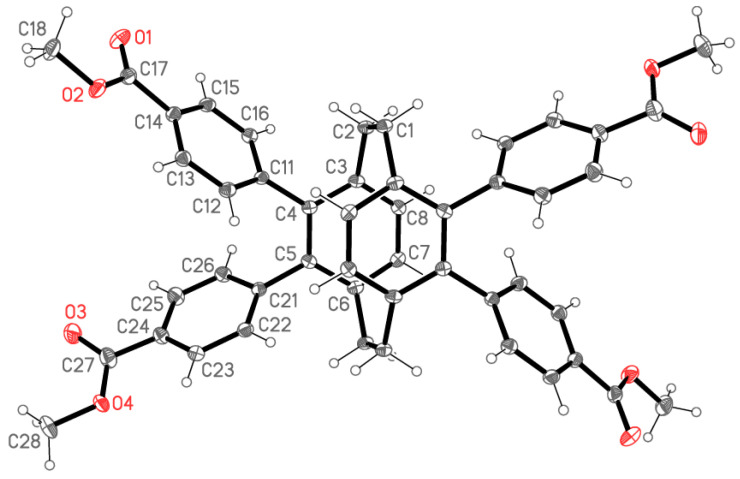
Structure of compound **14** in the crystal. Only the asymmetric unit is numbered.

The dicarboxylic acid **12** (Figure 5) crystallizes as the DMF disolvate, with inversion symmetry. The solvent molecules are involved in two hydrogen bonds with the carboxylic acid group, with one strong interaction O2–H02···O91 (H···O 1.65(2) Å) and one weaker interaction C93–H93···O1 (H···O 2.59 Å). The tetrasubstituted derivative **14** (Figure 6) also crystallizes with inversion symmetry.

**Figure 7:**
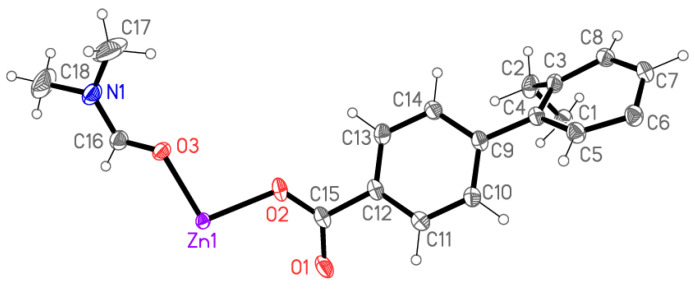
Structure of the asymmetric unit of compound **19** in the crystal.

**Figure 8:**
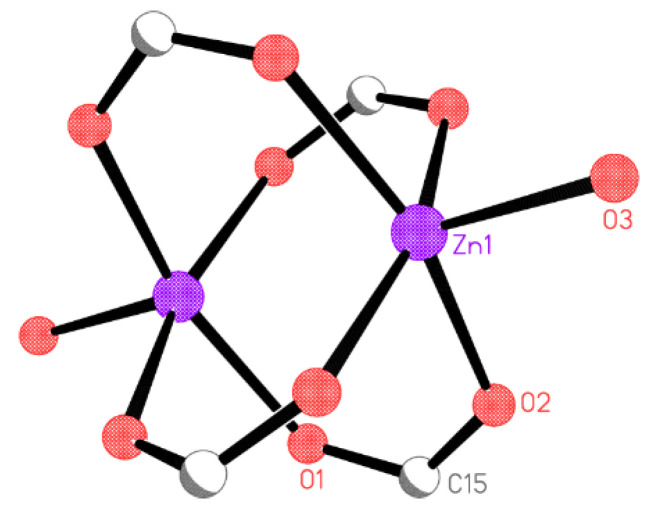
The paired coordination spheres of the zinc atoms in compound **19**.

**Figure 9:**
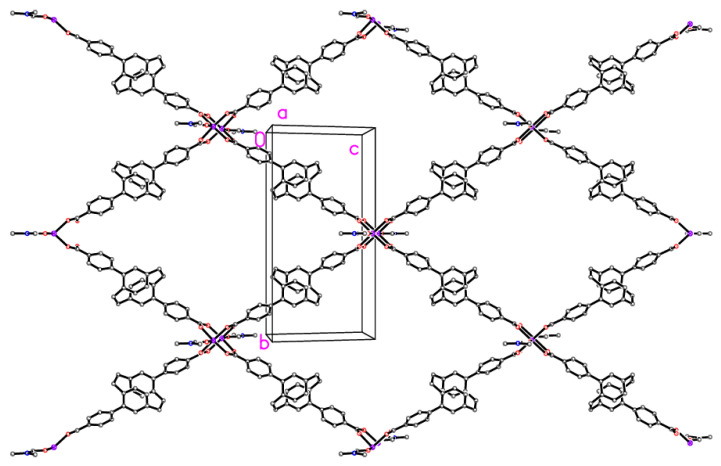
Structure of the two-dimensional polymer of compound **19**. Hydrogen atoms are omitted for clarity. Radii are arbitrary.

Upon reacting acid **12** with zinc nitrate in a mixture of DMF, ethanol and water, colorless crystals were obtained. Single crystal X-ray diffraction revealed that the polymeric zinc complex **19** has the overall composition Zn(cyclophane dicarboxylate)(DMF), whereby the zinc atom and the DMF lie in a crystallographic mirror plane, while the dicarboxylate displays inversion symmetry. The asymmetric unit is shown in Figure 7. The zinc center is five-coordinated in a square-pyramidal geometry, with a DMF oxygen atom at the apex and four carboxylate oxygen atoms in the base, which is exactly planar by symmetry; the zinc atom lies 0.3387(9) Å above this plane. Table 3 presents the relevant bond lengths and angles. The zinc coordination spheres are associated in pairs with overall 2/*m* symmetry (Figure 8), whereby the zinc atoms are only 2.9182(5) Å apart yet not formally bonded to each other. The extended structure is a two-dimensional coordination polymer (Figure 9) parallel to the plane (3 0 –1). The layers are staggered such that the DMF groups of one layer insert into the voids of the neighboring layers. It is worth mentioning that paddlewheel zinc-carboxylate motifs are fairly common and have been used before in coordination polymers.

Other metals, such as cadmium (II) and copper (II), were also used in reactions with **12**; however, no crystalline products were obtained.

## 4. Conclusions

The synthesis and characterization of twelve di- and tetracarboxylic [2.2]paracyclophane derivatives were accomplished via Suzuki coupling between the corresponding brominated [2.2]paracyclophanes and 4-(methoxycarbonyl)phenylboronic acid. These compounds represent valuable building blocks for coordination polymers. In one such example, a 2D coordination polymer comprising zinc-carboxylate paddlewheel clusters linked by cyclophane cores was obtained. Furthermore, these polyfunctionalized [2.2]paracyclophanes could also prove interesting for other related fields, such as polymer or covalent organic framework design [27].

## Figures and Tables

**Table 1:** Crystallographic data and structure refinement details for compounds **5**–**8**.

Compound	5	6	7	8
CCDC number	2245482	2245483	2245484	2245485
Formula	C_32_H_28_O_4_	C_32_H_28_O_4_	C_32_H_28_O_4_	C_32_H_28_O_4_
*M*_r_	476.54	476.54	476.54	476.54
Crystal size (mm)	0.2 × 0.2 × 0.04	0.2 × 0.15 × 0.15	0.22 × 0.17 × 0.17	0.2 × 0.2 × 0.05
Crystal system	orthorhombic	triclinic	triclinic	triclinic
Space group	*Pca*2_1_	*P*(−1)	*P*(−1)	*P*(−1)
Temperature (°C)	−173	−173	−173	−173
*a* (Å)	26.8431(5)	10.1243(2)	6.9793(2)	6.88619(13)
*b* (Å)	7.86457(15)	10.3597(3)	9.3525(3)	8.94644(16)
*c* (Å)	11.1615(2)	12.7502(3)	18.0147(6)	9.47904(18)
α (°)	90	67.824(2)	92.488(2)	92.4255(14)
β (°)	90	75.462(2)	91.462(2)	94.0838(16)
γ (°)	90	81.498(2)	93.942(2)	92.4901(14)
*V* (Å^3^)	2356.29	1196.62	1171.52	581.34
*Z*	4	2	2	1
*D*_x_ (Mg m^−3^)	1.343	1.323	1.351	1.361
λ (Å)	0.71073 (Mo *K*α)	0.71073 (Mo *K*α)	0.71073 (Mo *K*α)	0.71073 (Mo *K*α)
μ (mm^−1^)	0.09	0.09	0.09	0.09
Transmissions	0.808–1.000	0.927–1.000	0.982–1.000	0.801–1.000
*F*(000)	1008	504	504	252
2θ_max_	78.7	82.6	76.7	82.7
Refl. measured	175,596	153,237	17,398	74,728
Refl. indep.	13,671	15,664	17,398	7643
*R*_int_	0.044	0.029	n/a	0.25
Parameters	351	351	352	176
Restraints	16	15	15	3
*wR*(*F*^2^, all refl.)	0.092	0.117	0.131	0.123
*R*(*F*, >4σ(*F*))	0.034	0.037	0.045	0.039
*S*	1.06	1.06	1.10	1.04
Max. Δ*p* (e Å^−3^)	0.48, −0.20	0.59, −0.28	0.53, −0.24	0.61, −0.38

**Table 2:** Crystallographic data and structure refinement details for compounds **12·2DMF**, **14** and **19**.

Compound	12·2DMF	14	19
CCDC number	2245486	2245487	2245488
Formula	C_36_H_38_N_2_O_6_	C_48_H_40_O_8_	C_33_H_29_NO_5_Zn
*M*_r_	594.68	392.43	584.94
Crystal size (mm)	0.2 × 0.15 × 0.1	0.2 × 0.15 × 0.1	0.08 × 0.04 × 0.005
Crystal system	monoclinic	monoclinic	monoclinic
Space group	*P*2_1_/*c*	*C*2/*c*	*I*2/*m*
Temperature (°C)	−173	−173	−173
*a* (Å)	17.3701(4)	35.9642(3)	10.44383(15)
*b* (Å)	6.96876(14)	13.38270(12)	22.4818(3)
*c* (Å)	13.2806(3)	7.62745(7)	11.21302(17)
α (°)	90	90	90
β (°)	108.452(2)	93.7660(8)	99.6841(14)
γ (°)	90	90	90
*V* (Å^3^)	1524.95	3663.15	2595.26
*Z*	2	4	4
*D*_x_ (Mg m^−3^)	1.295	1.350	1.497
λ (Å)	0.71073 (Mo *K*α)	1.54184 (Cu *K*α)	1.54184 (Cu *K*α)
μ (mm^−1^)	0.09	0.74	1.7
Transmissions	0.780–1.000	0.709–1.000	0.752–1.000
*F*(000)	632	1568	1216
2θ_max_	80.5	155.2	155.0
Refl. measured	149,470	89,000	62,900
Refl. indep.	9604	3893	2834
*R*_int_	0.047	0.054	0.033
Parameters	230	292	203
Restraints	19	24	3
*wR*(*F*^2^, all refl.)	0.116	0.116	0.069
*R*(*F*, >4σ(*F*))	0.037	0.051	0.028
*S*	1.04	1.18	1.06
Max. Δ*p* (e Å^−3^)	0.61, −0.29	0.31, −0.25	0.34, −0.52

**Table 3:** Bond lengths (Å) and angles (°) at the zinc atom of compound **19**.

Zn(1)-O(3)	1.9879(14)
Zn(1)-O(2)#1	2.0356(10)
Zn(1)-O(2)	2.0356(10)
Zn(1)-O(1)#2	2.0499(11)
Zn(1)-O(1)#3	2.0499(11)
Zn(1)···Zn(1)#3	2.9182(5)
O(3)-Zn(1)-O(2)#1	98.89(4)
O(3)-Zn(1)-O(2)	98.89(4)
O(2)#1-Zn(1)-O(2)	85.64(6)
O(3)-Zn(1)-O(1)#2	100.21(5)
O(2)#1-Zn(1)-O(1)#2	92.66(5)
O(2)-Zn(1)-O(1)#2	160.85(5)
O(3)-Zn(1)-O(1)#3	100.21(5)
O(2)#1-Zn(1)-O(1)#3	160.85(5)
O(2)-Zn(1)-O(1)#3	92.67(5)
O(1)#2-Zn(1)-O(1)#3	82.71(7)

Symmetry transformations used to generate equivalent atoms: #1 *x*, –*y* + 1, *z*; #2 –*x* + 2, –*y* + 1, –*z* + 2; #3 –*x* + 2, *y*, –*z* + 2.

## Data Availability

The CIF files for structures **5**–**8**, **12**, **14** and **19** were uploaded to the Cambridge Crystallographic Data Centre and can be accessed free of charge at https://www.ccdc.cam.ac.uk/structures/.

## Supplementary Materials

The following supporting information can be downloaded at https://www.mdpi.com/article/10.3390/ma16114051/s1, Figures S1–S24: ^1^H and ^13^C NMR spectra of **5**–**12**, **14**, **15**, **17** and **18**; Figures S25–S37: FT-IR spectra of **5**–**12**, **14**, **15**, **17, 18** and **19**.
