# Supplementary material for: [2.2]Paracyclophane Derivatives as Building Blocks for Coordination Polymers
Source: Materials (Basel). 2023 May 29;16(11):4051. doi: 10.3390/ma16114051 (PMC10254932; doi:10.3390/ma16114051)
Supplement: Supplementary file 1 [file materials-16-04051-s001.zip › materials-2415805-supplementary.pdf]

Supporting Information

for

[2.2]Paracyclophane derivatives as coordination polymer building blocks

Mihail Lucian Birsa [1], Henning Hopf [1,2], Peter George Jones [1,3], Laura Gabriela Sarbu [1,*] and Lucian Gabriel Bahrin [1,4,*]

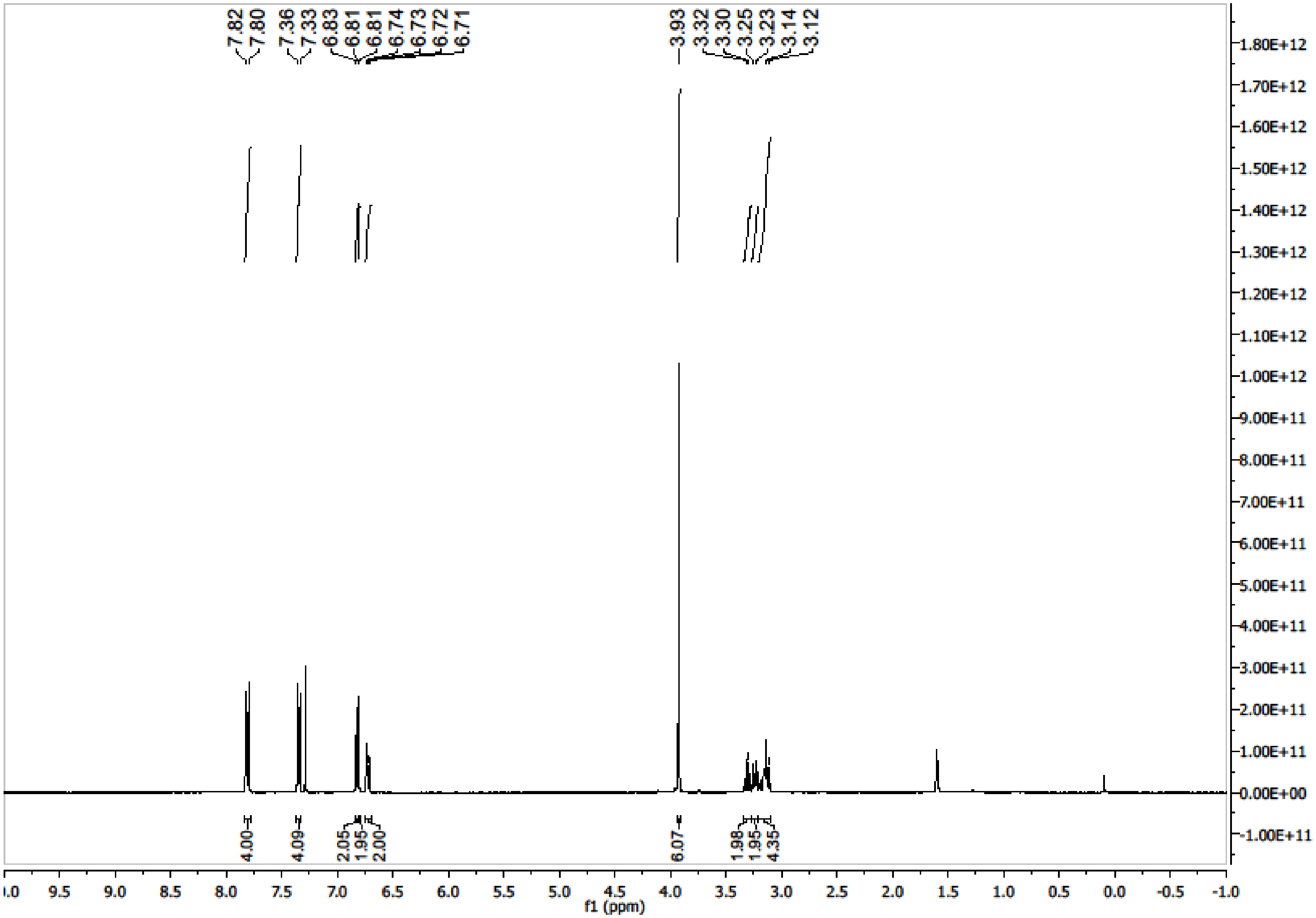

Figure S1 – The ^{1}H NMR spectrum of **5**.

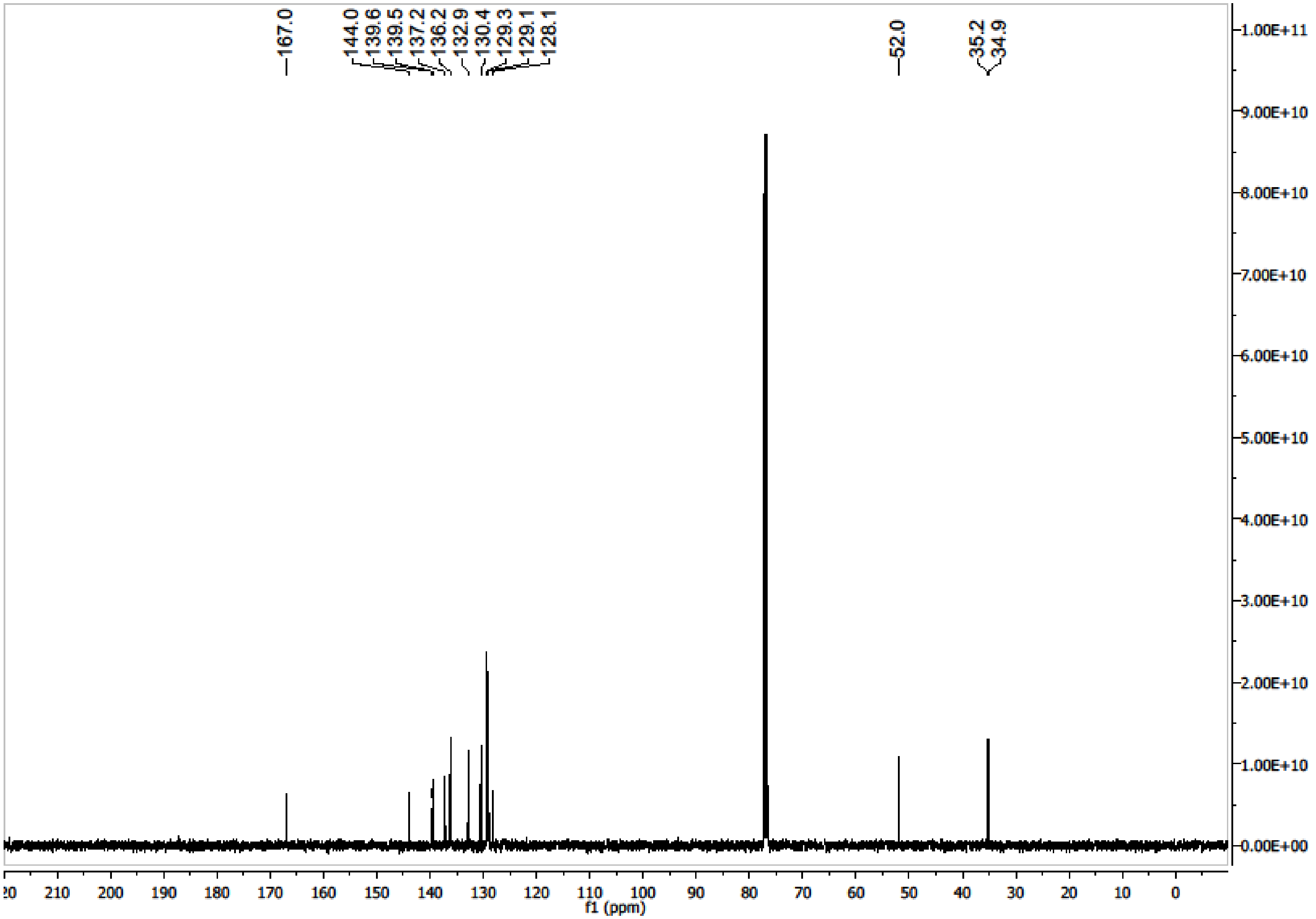

Figure S2 – The ^{13}C NMR spectrum of **5**.

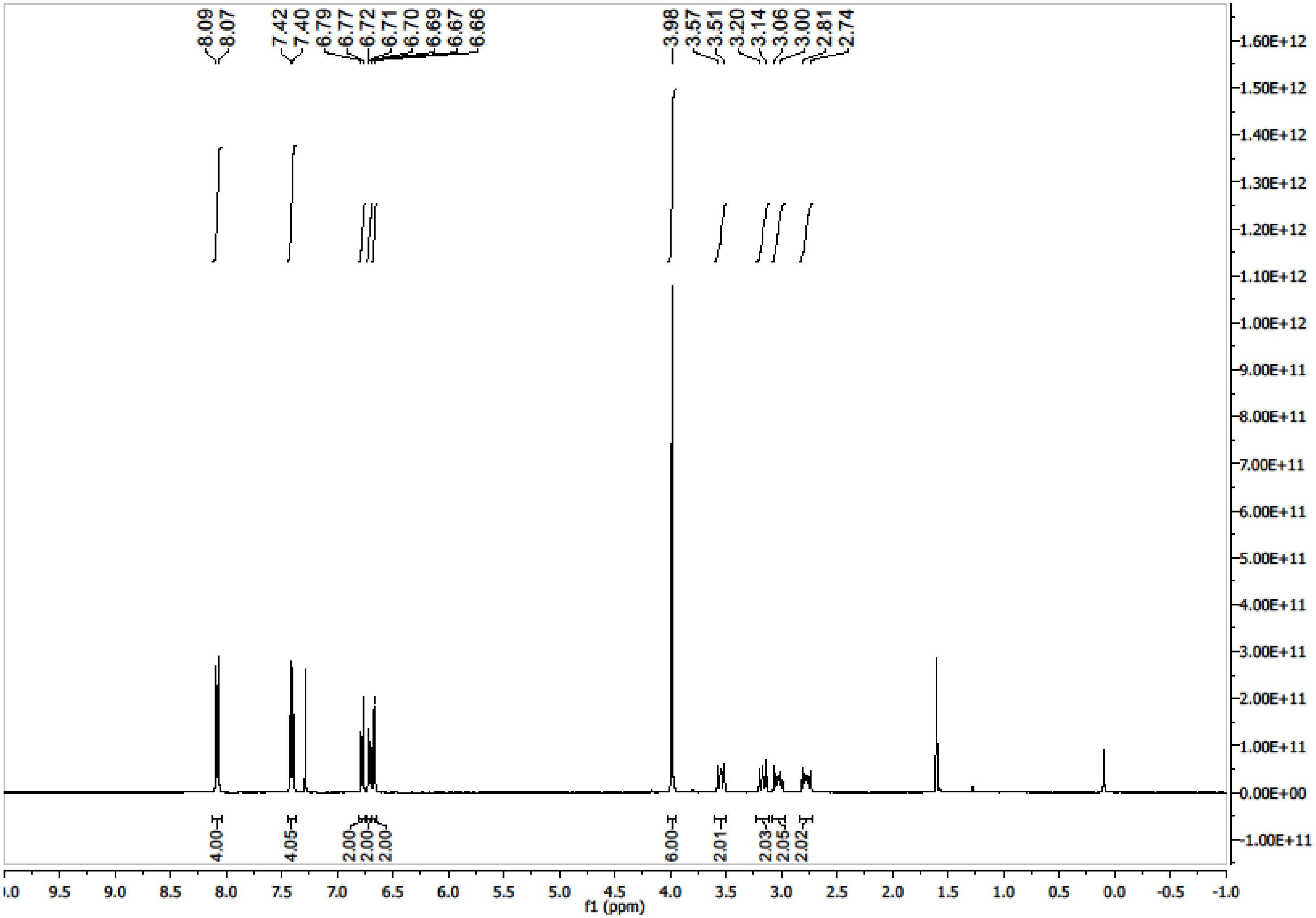

Figure S3 – The ^{1}H NMR spectrum of **6**.

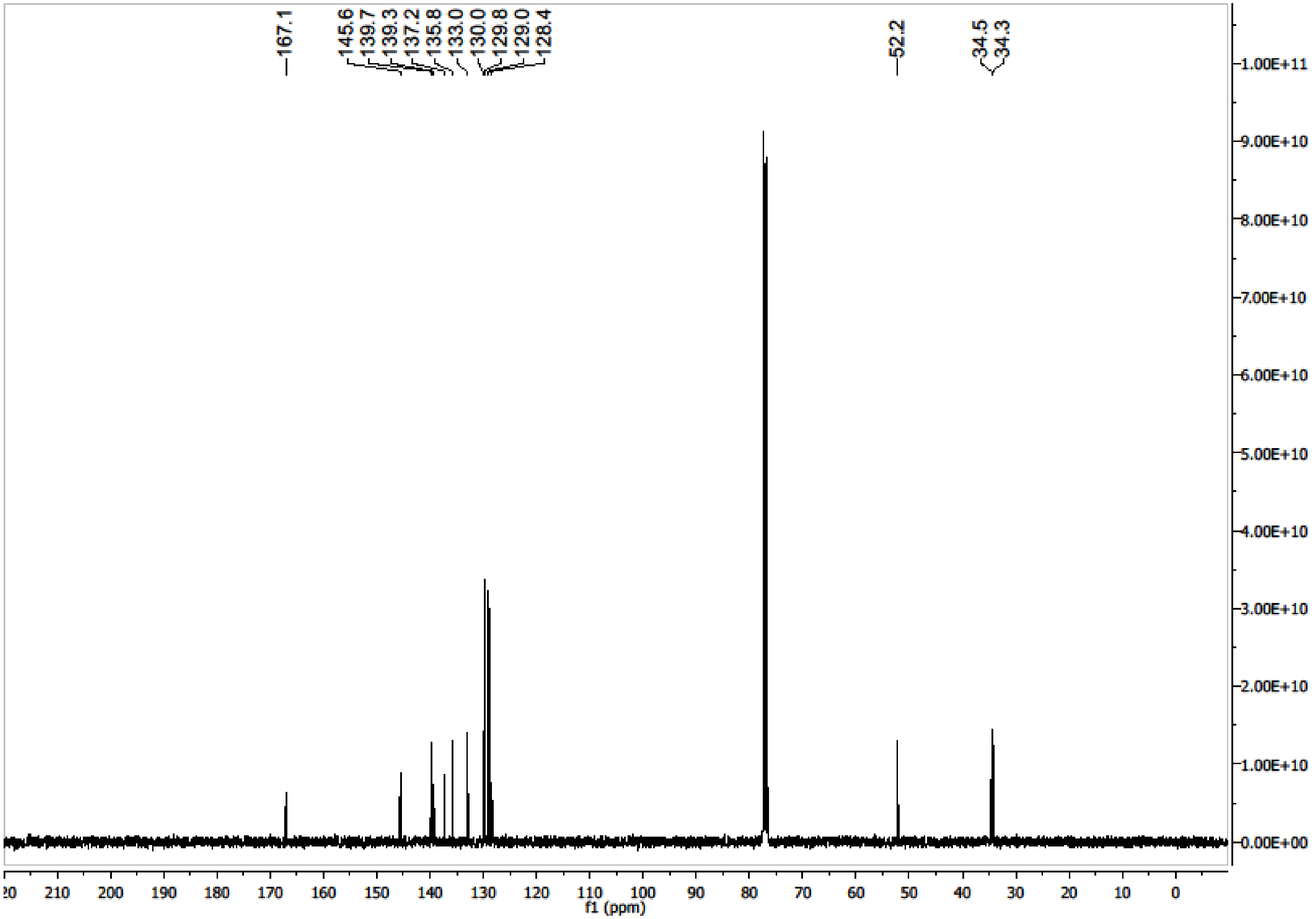

Figure S4 – The ^{13}C NMR spectrum of **6**.

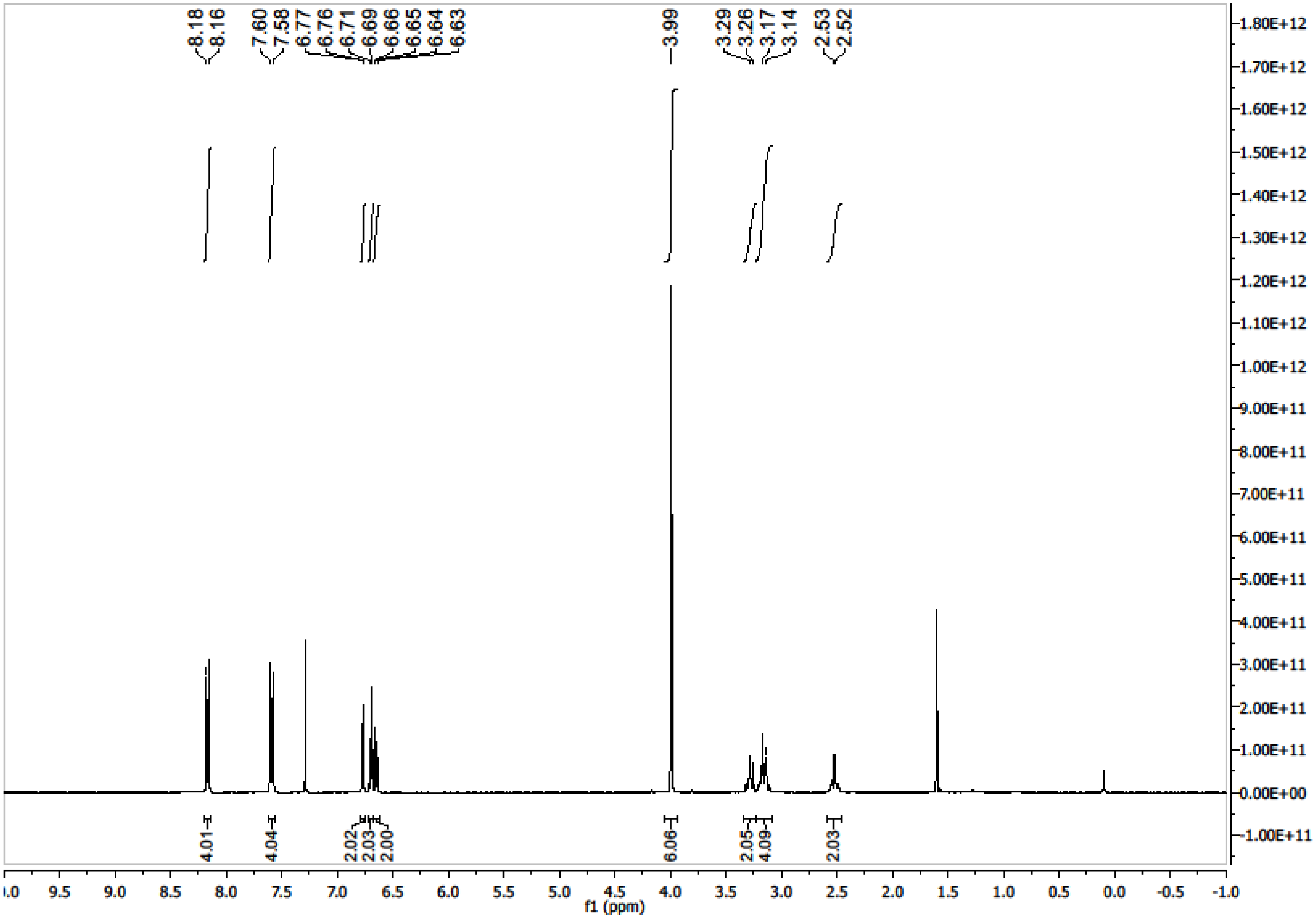

Figure S5 – The ^{1}H NMR spectrum of **7**.

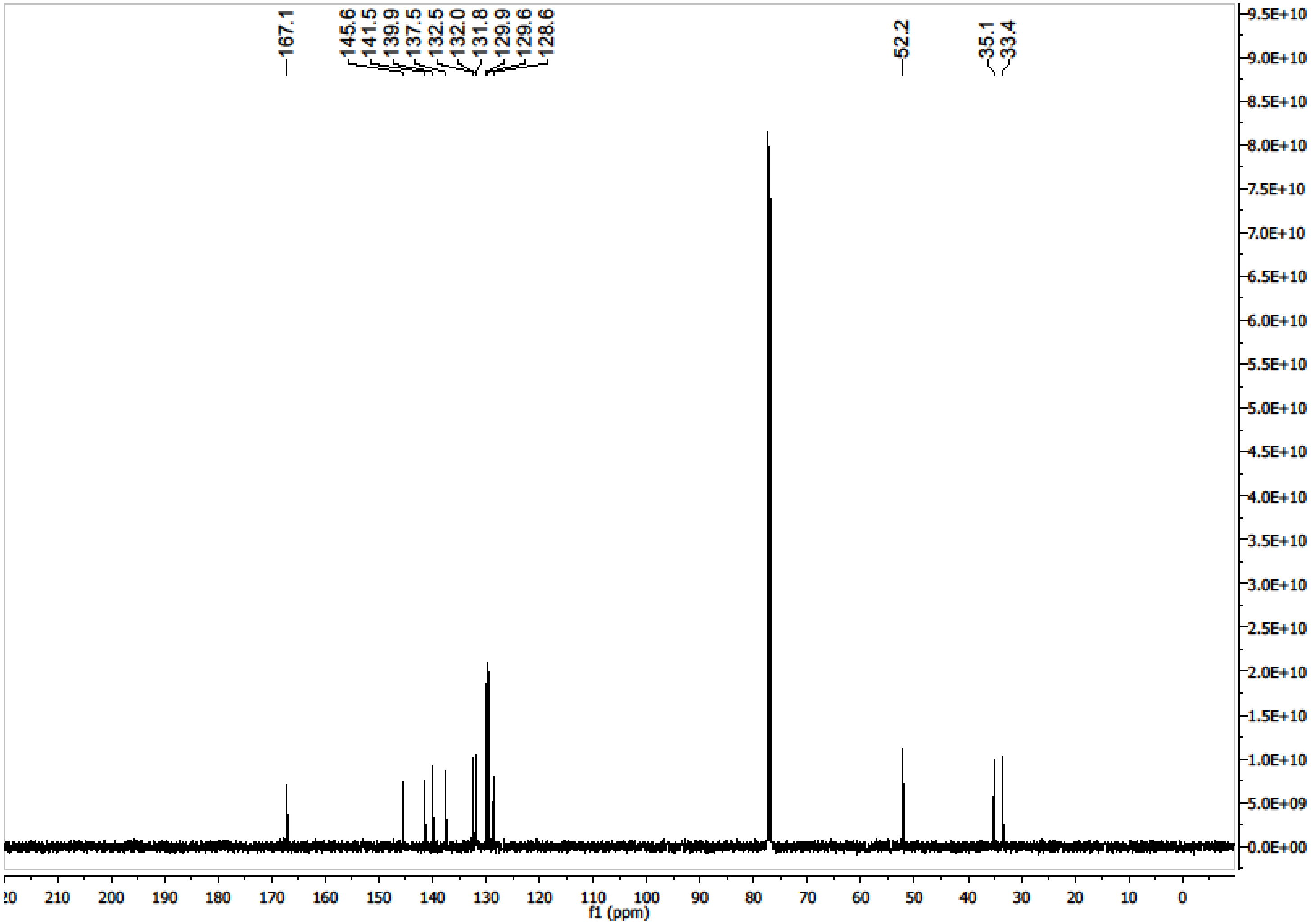

Figure S6 – The ^{13}C NMR spectrum of **7**.

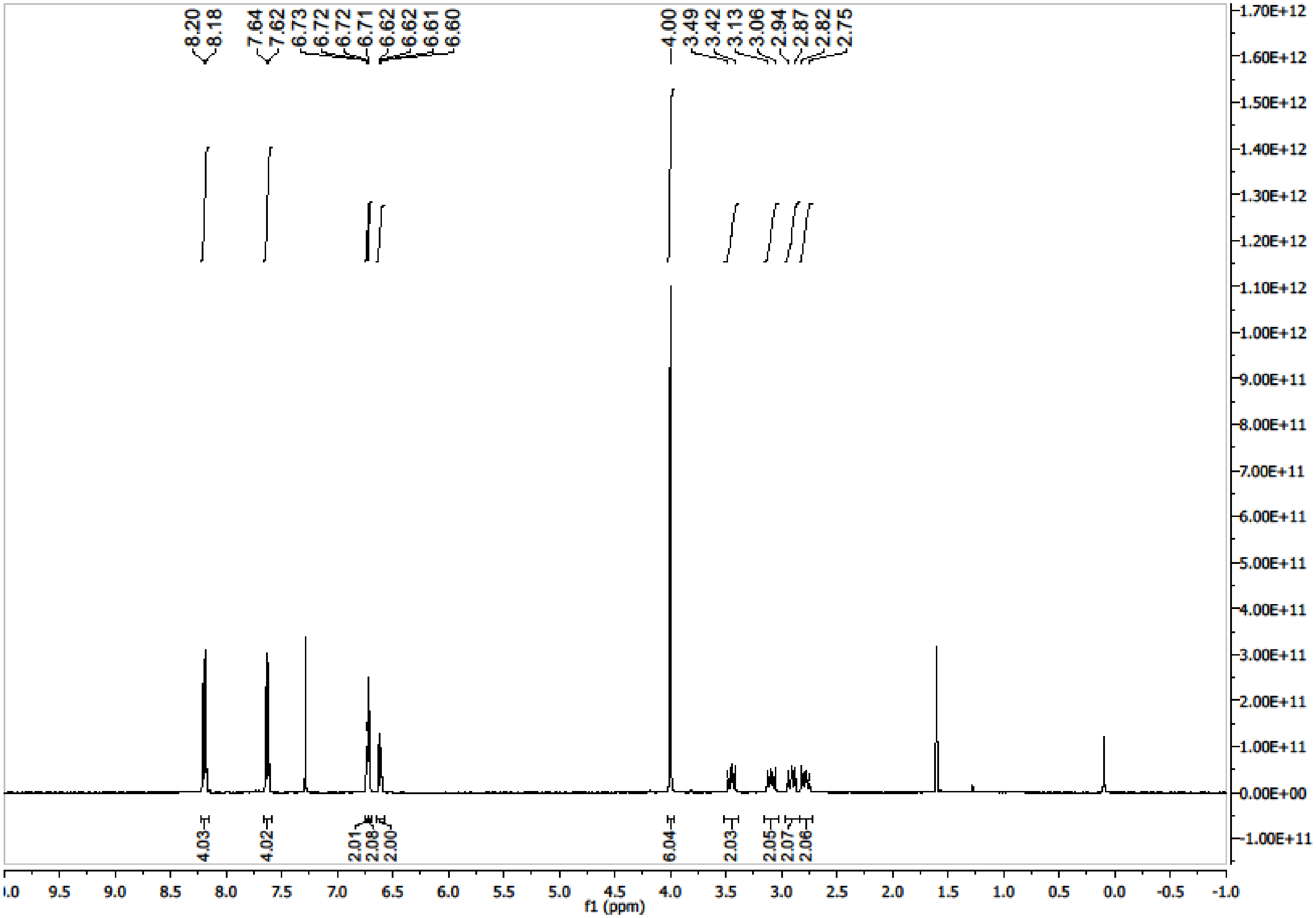

Figure S7 – The ^{1}H NMR spectrum of **8**.

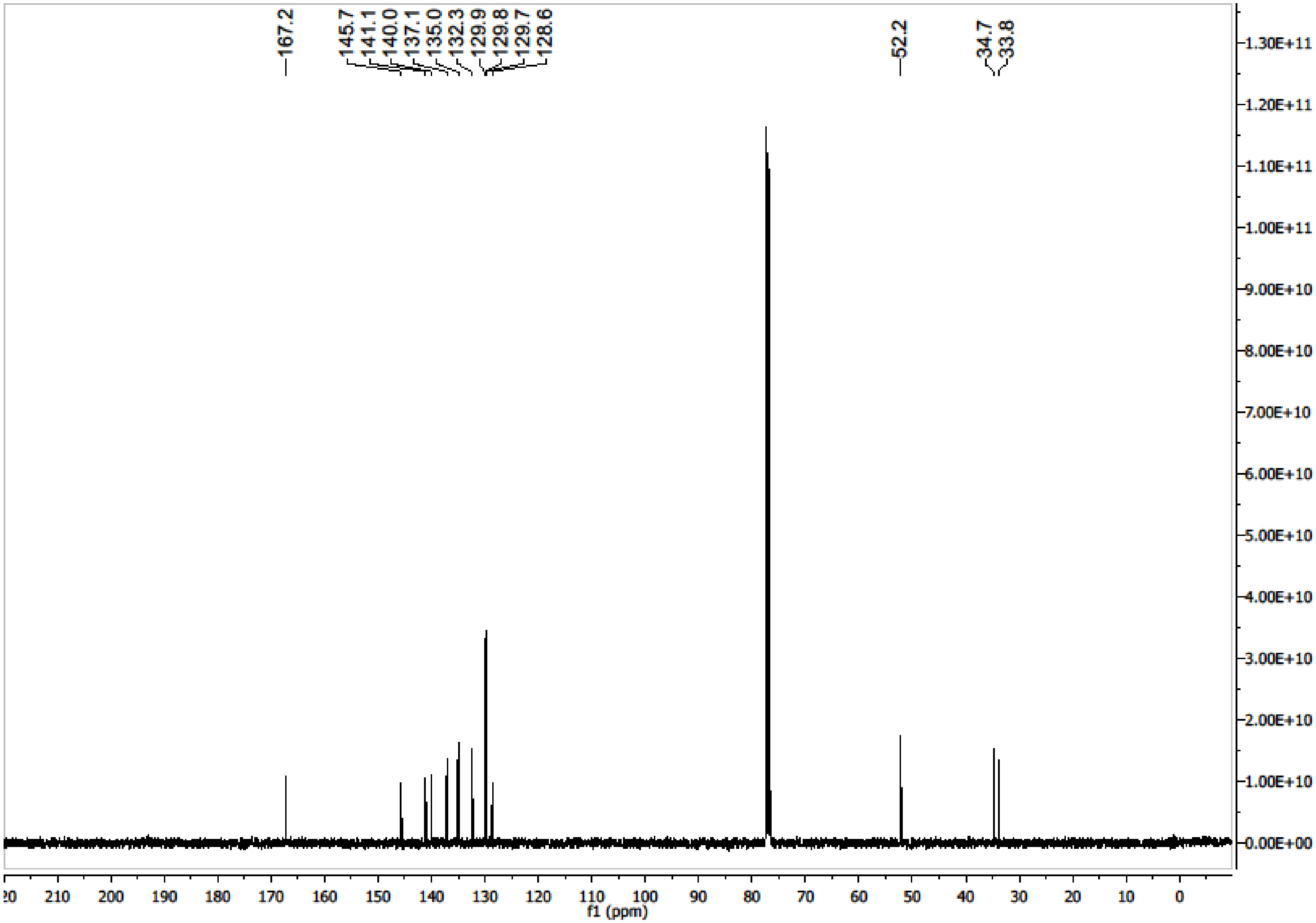

Figure S8 – The ^{13}C NMR spectrum of **8**.

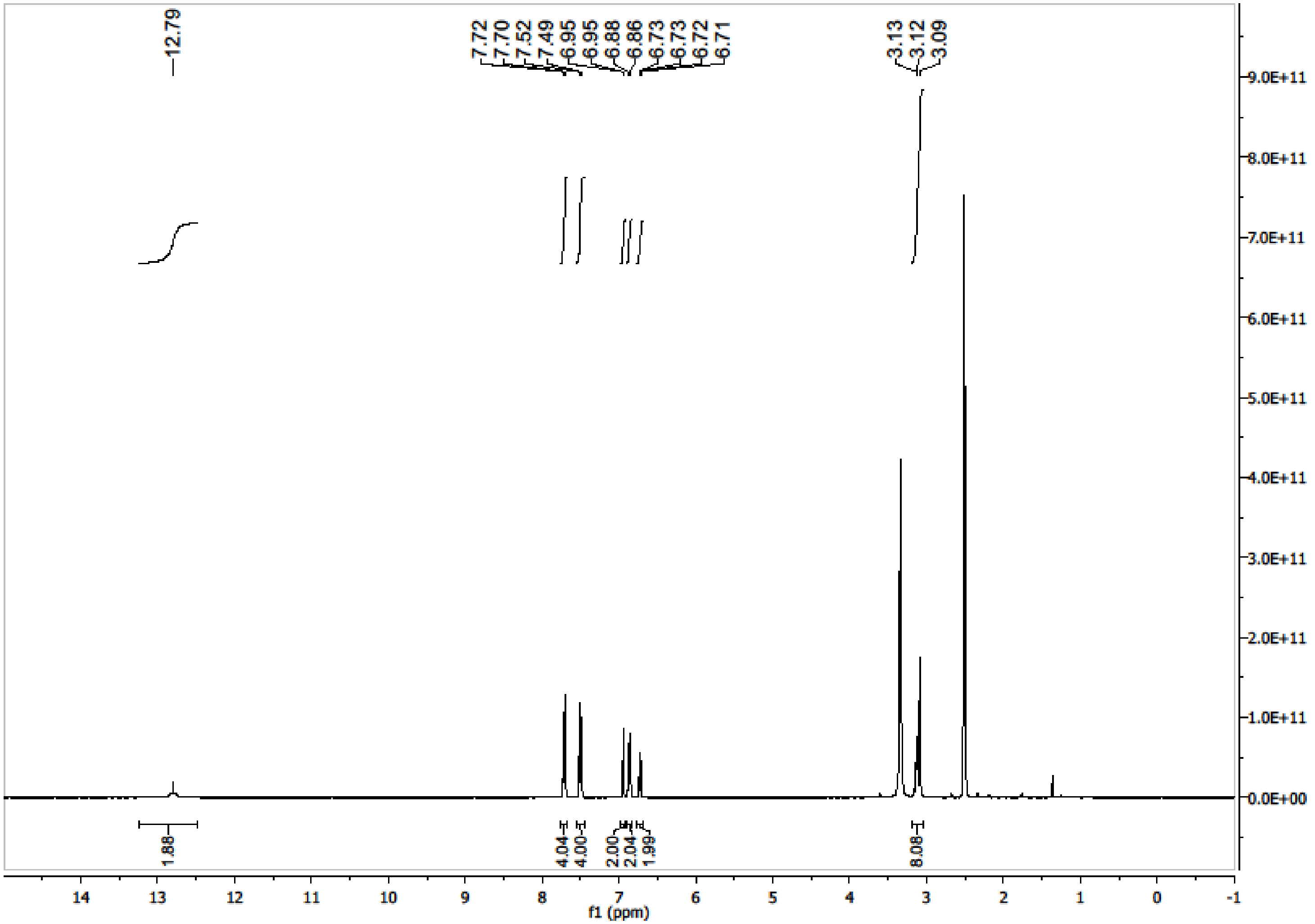

Figure S9 – The ^{1}H NMR spectrum of **9**.

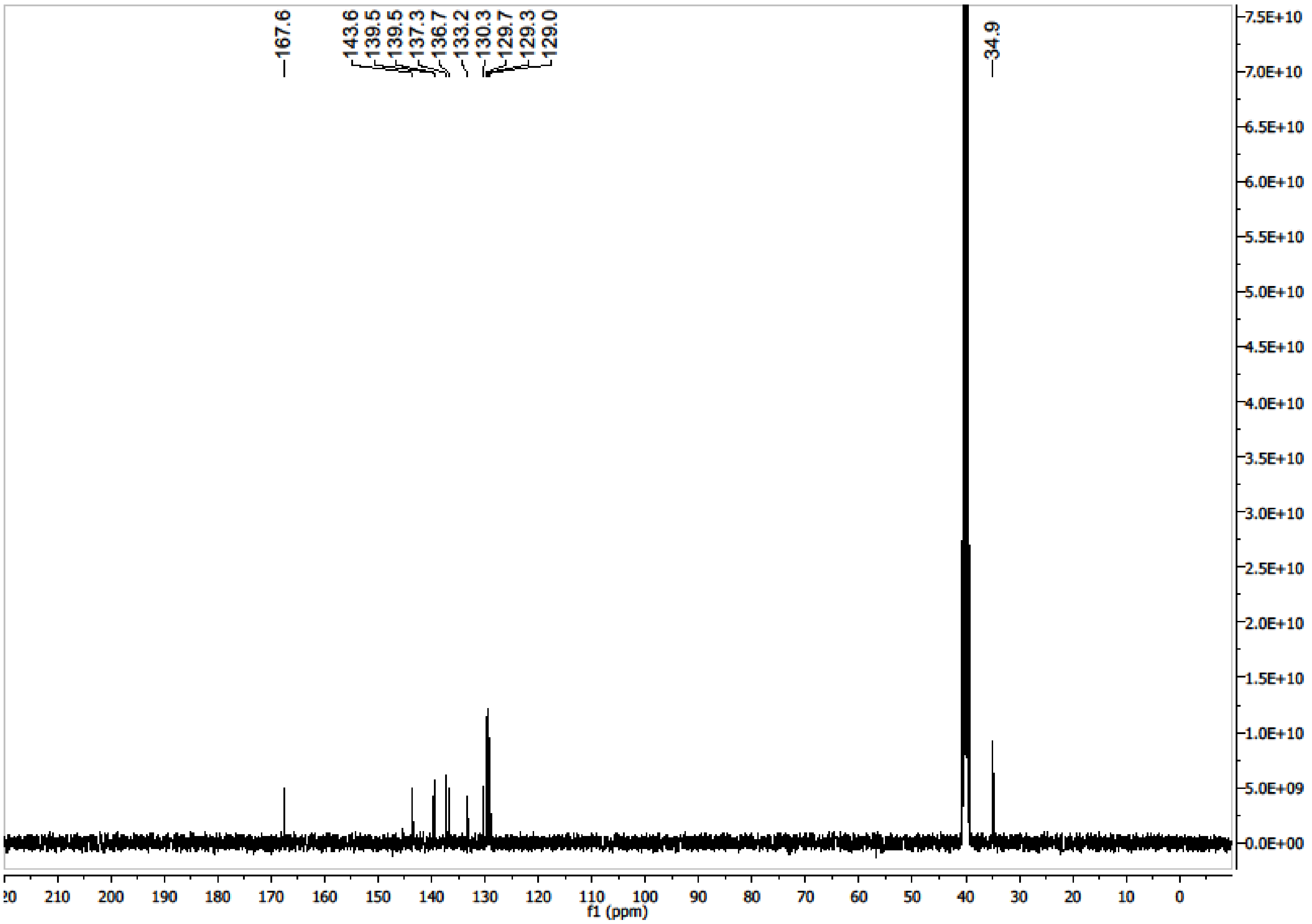

Figure S10 – The ^{13}C NMR spectrum of **9**.

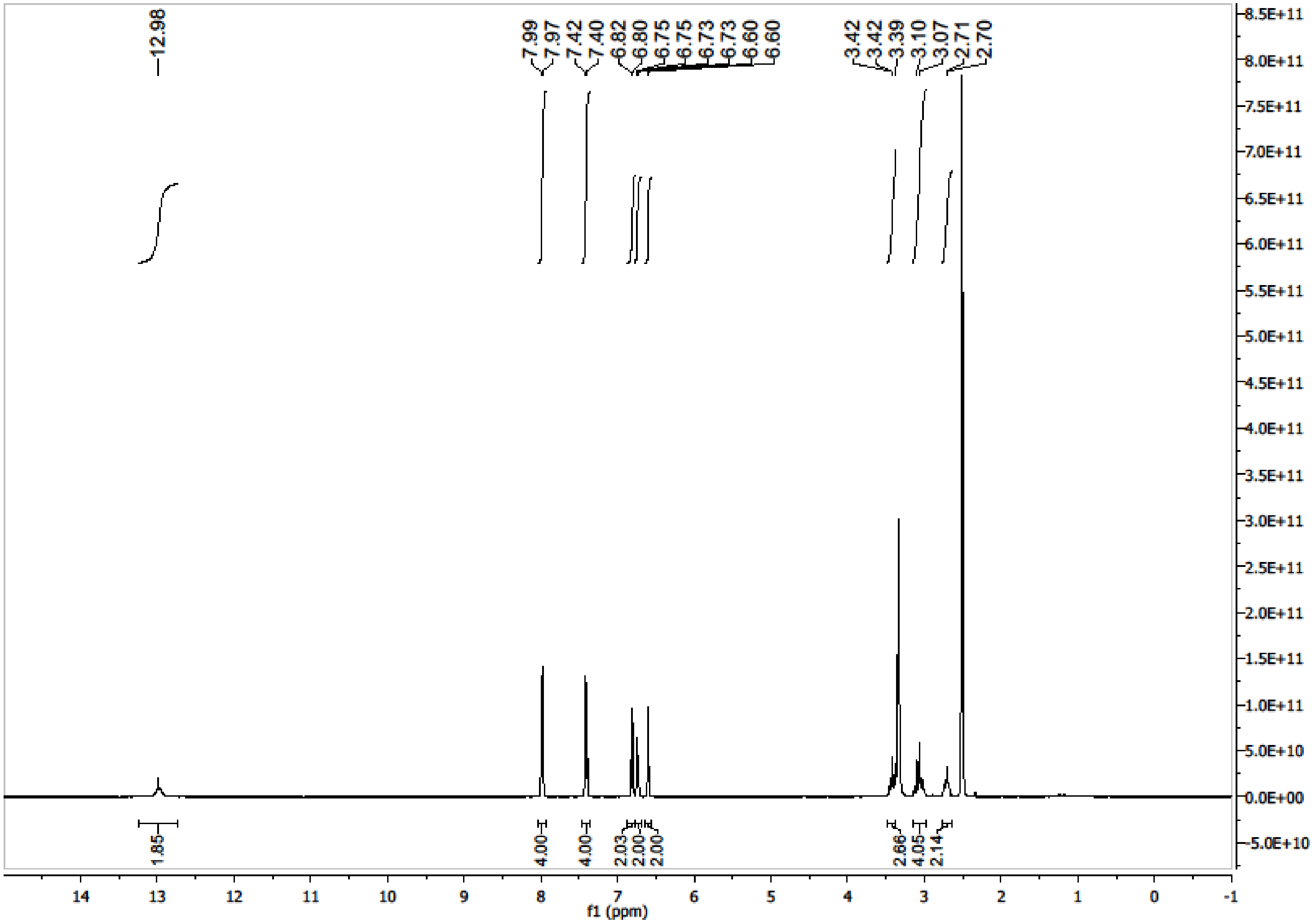

Figure S11 – The ^{1}H NMR spectrum of **10**.

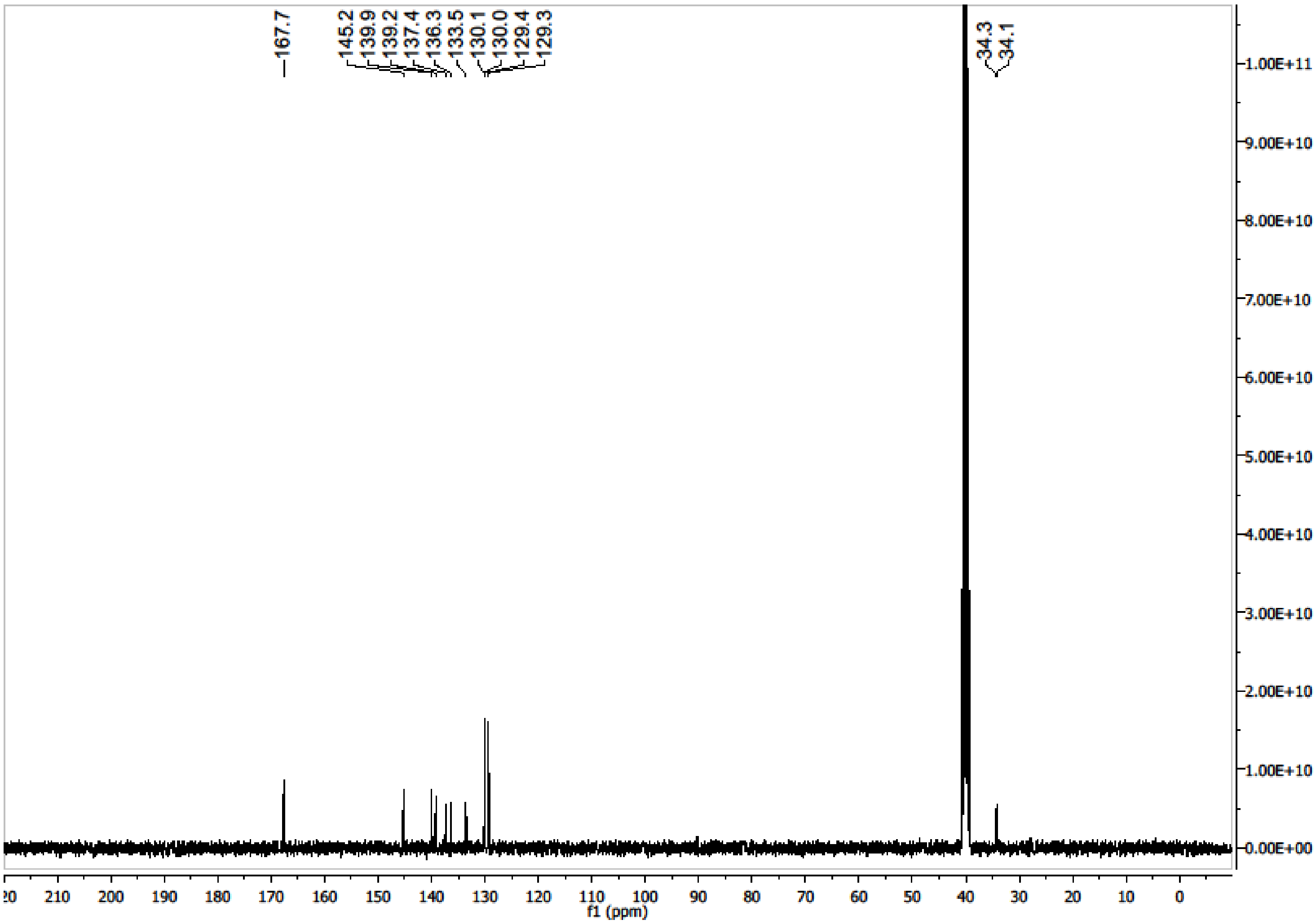

Figure S12 – The ^{13}C NMR spectrum of **10**.

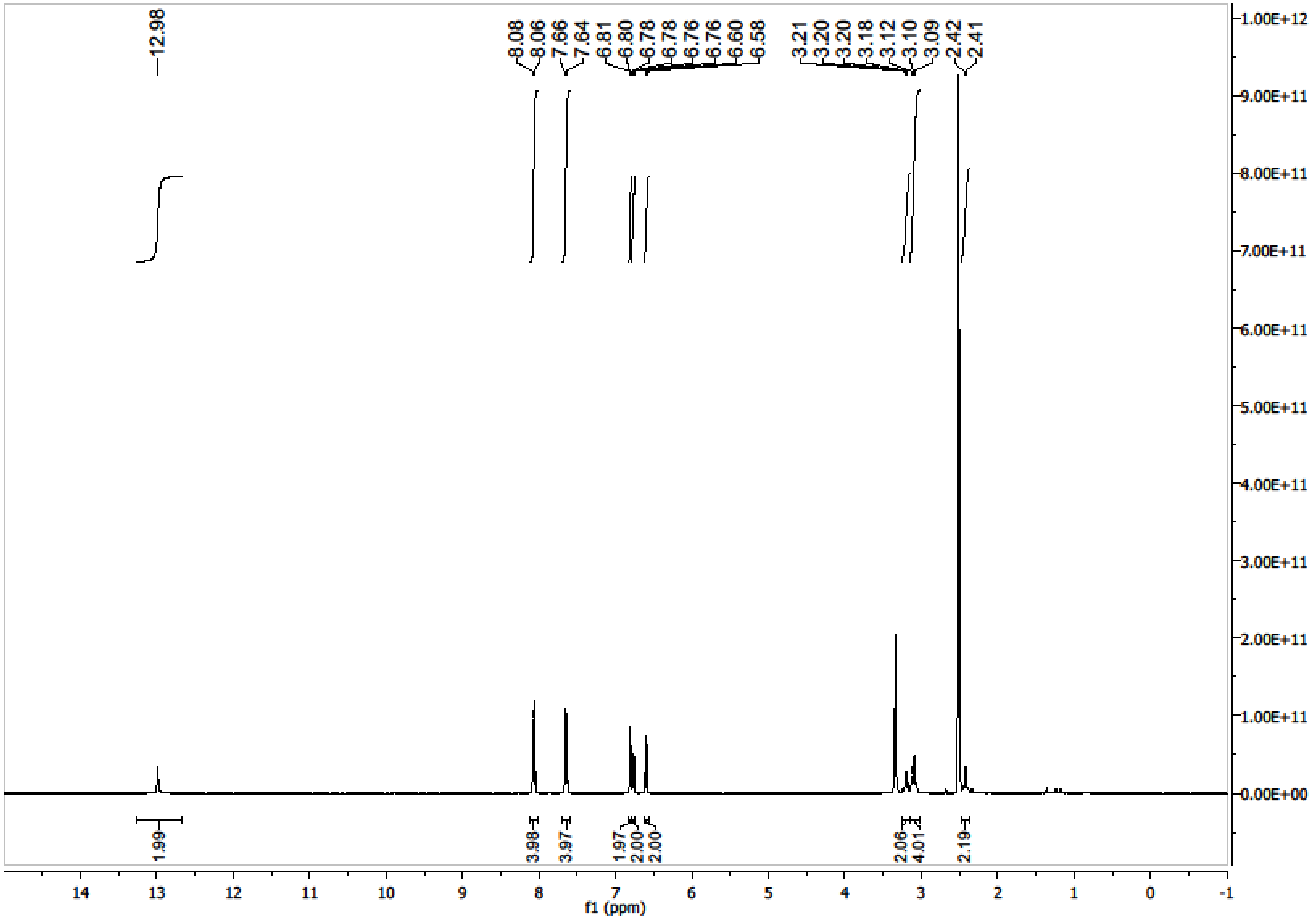

Figure S13 – The ^{1}H NMR spectrum of **11**.

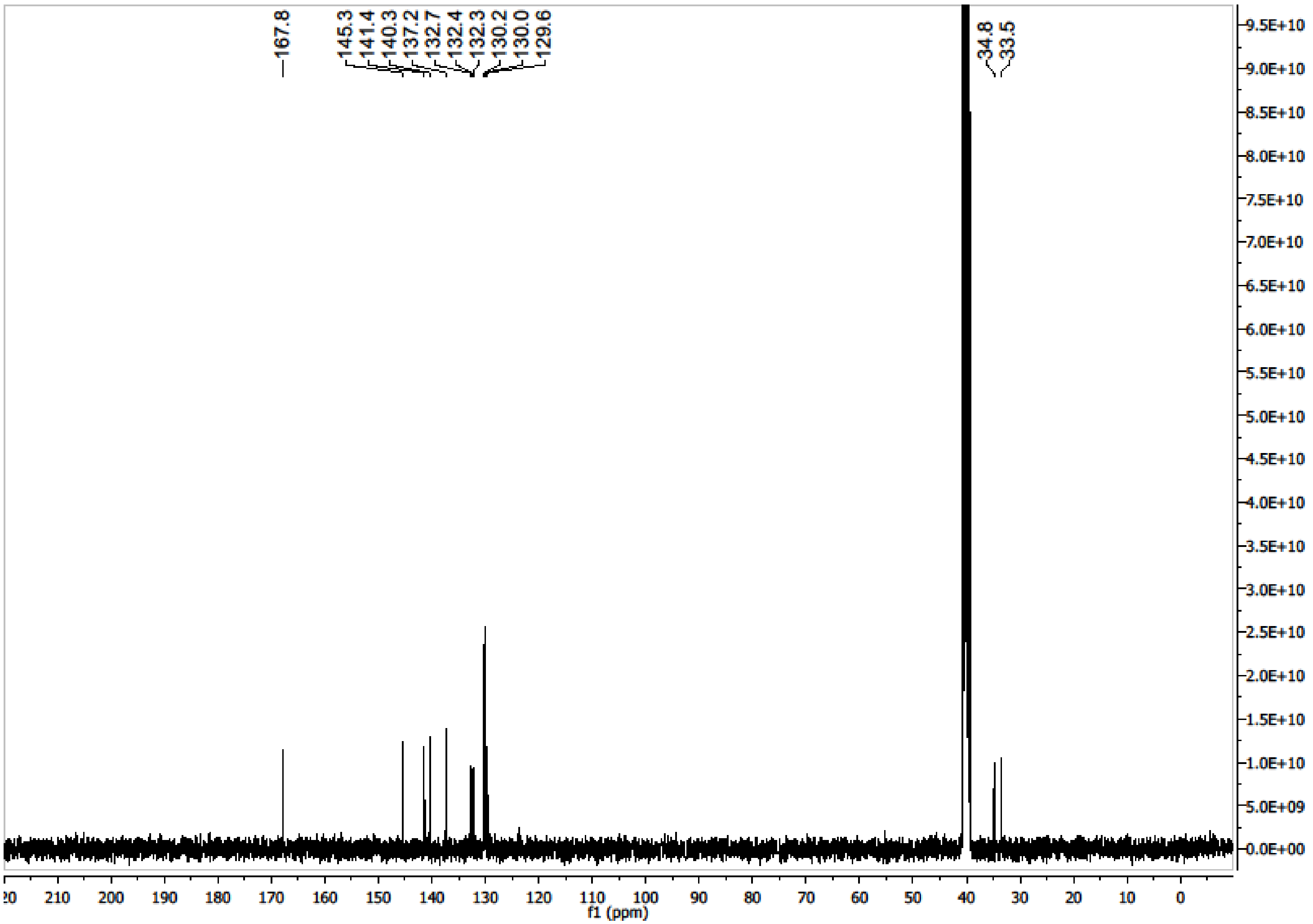

Figure S14– The ^{13}C NMR spectrum of **11**.

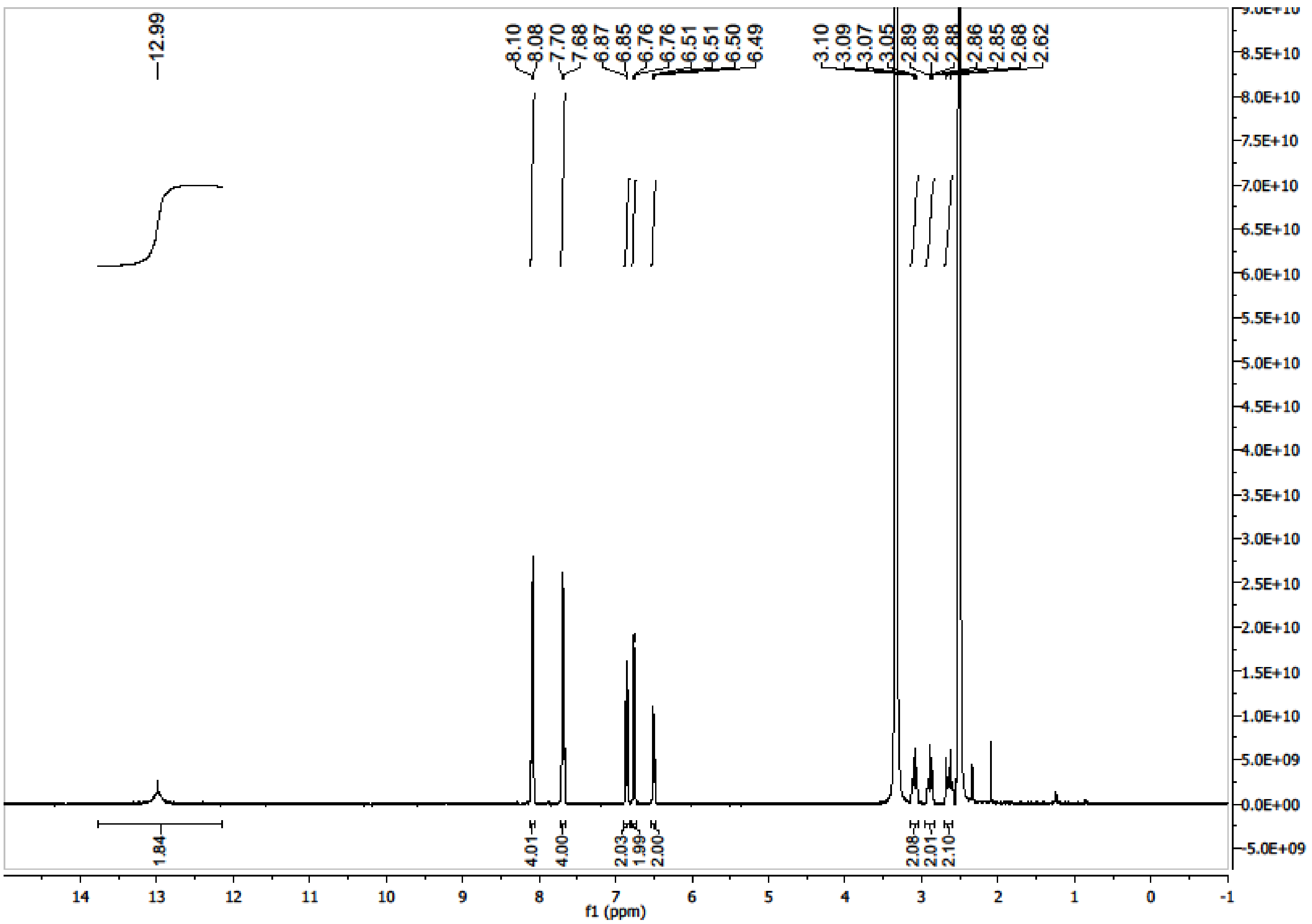

Figure S15– The ^{1}H NMR spectrum of **12**.

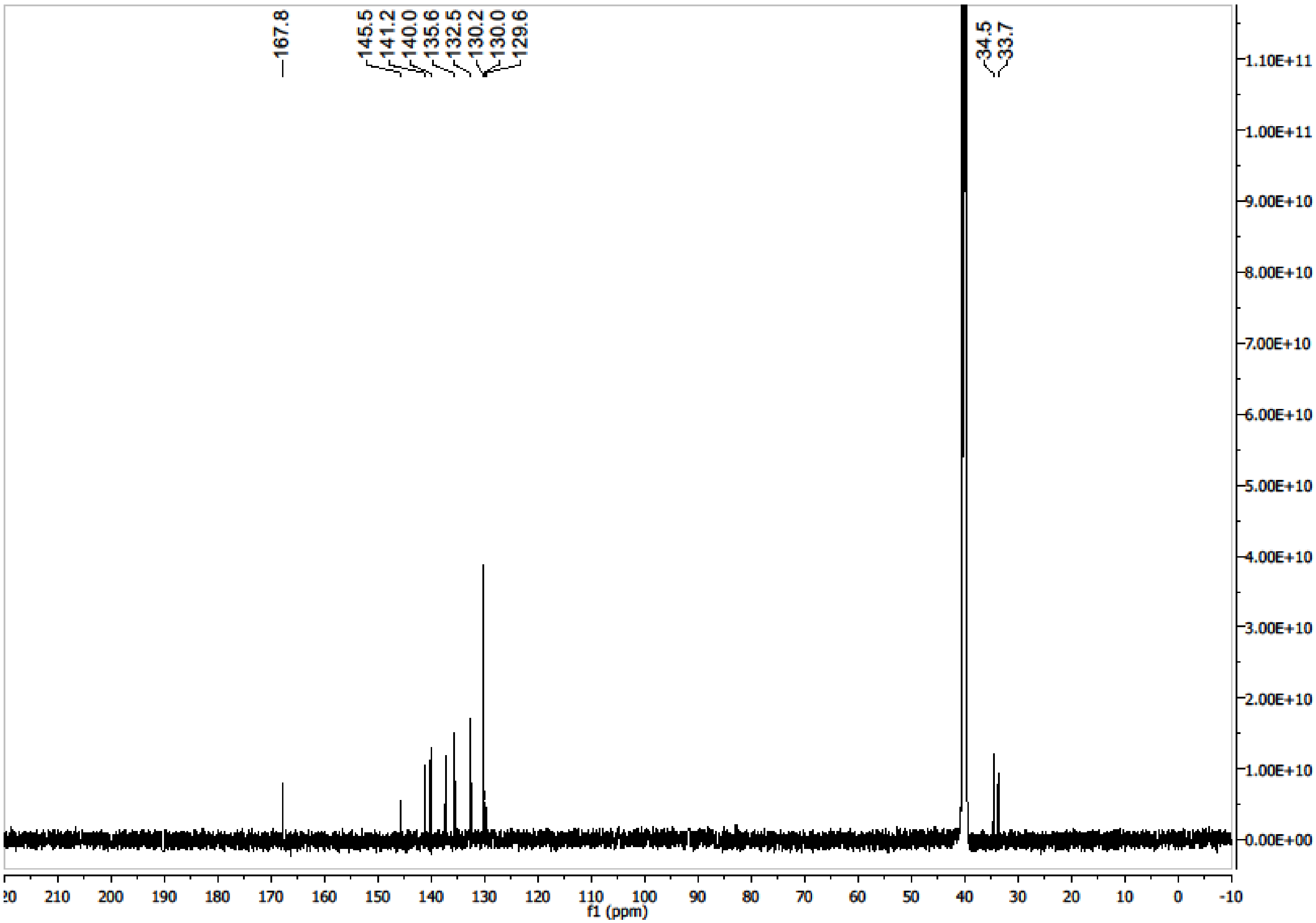

Figure S16– The ^{13}C NMR spectrum of **12**.

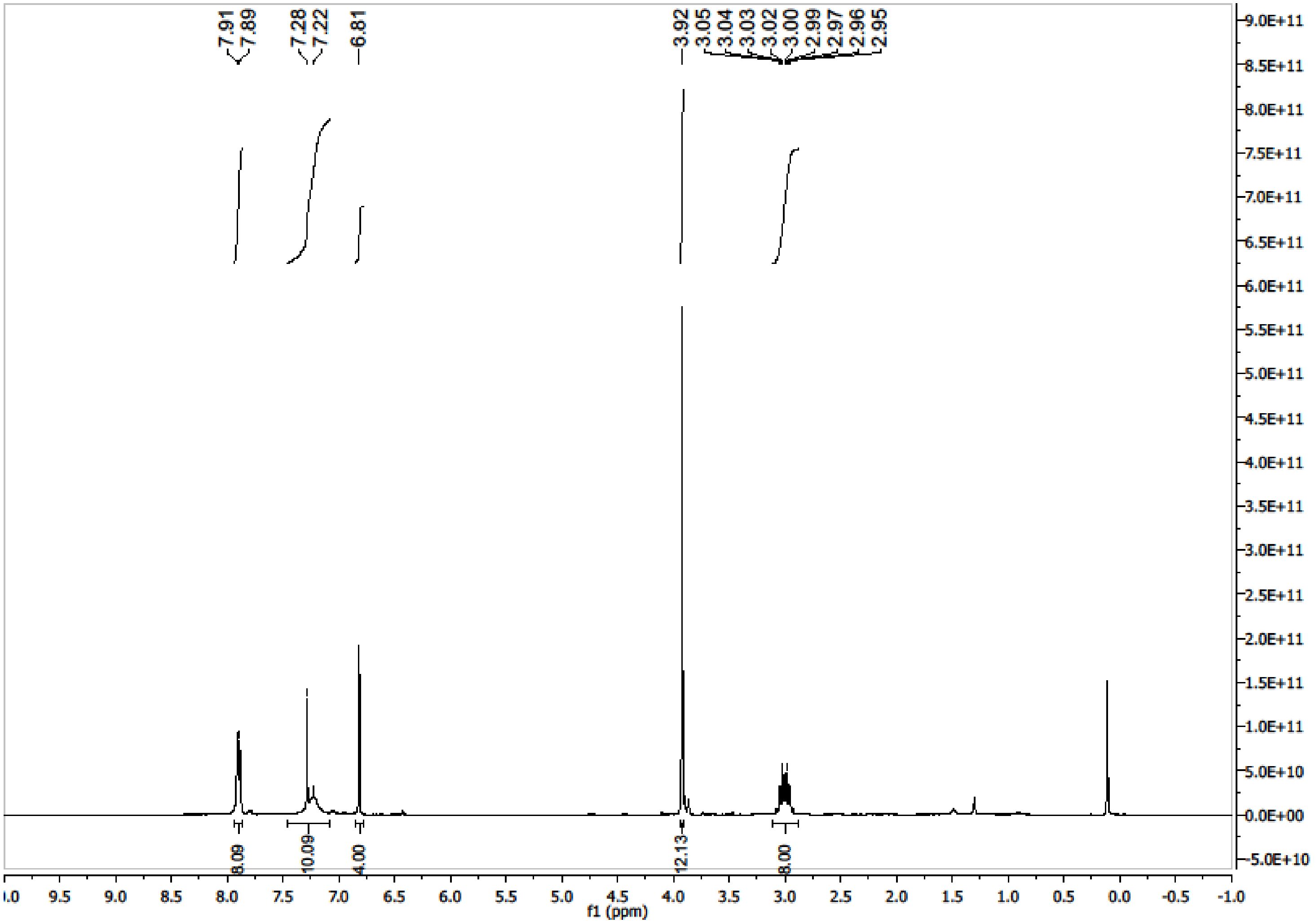

Figure S17– The ^{1}H NMR spectrum of **14**.

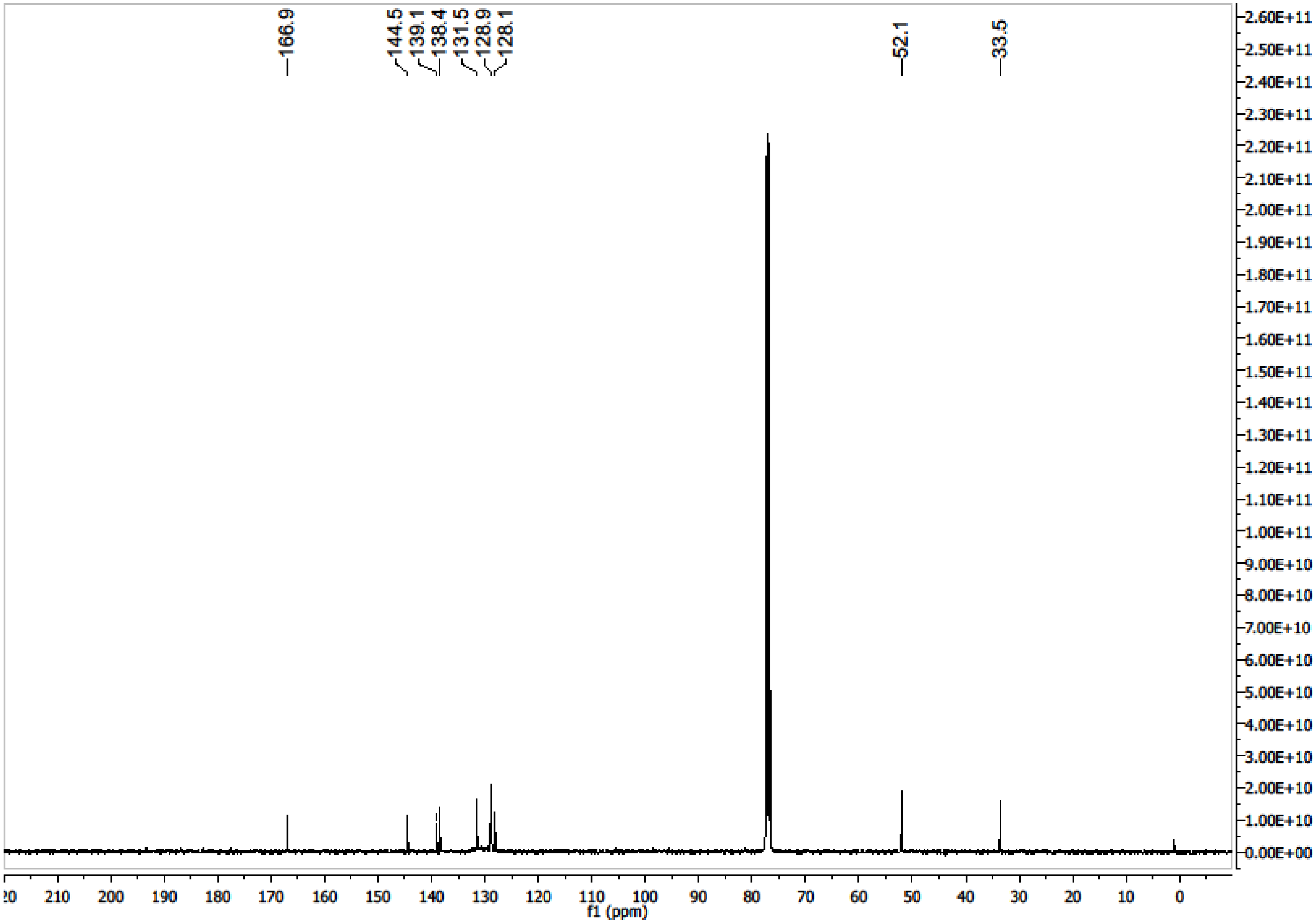

Figure S18– The ^{13}C NMR spectrum of **14**.

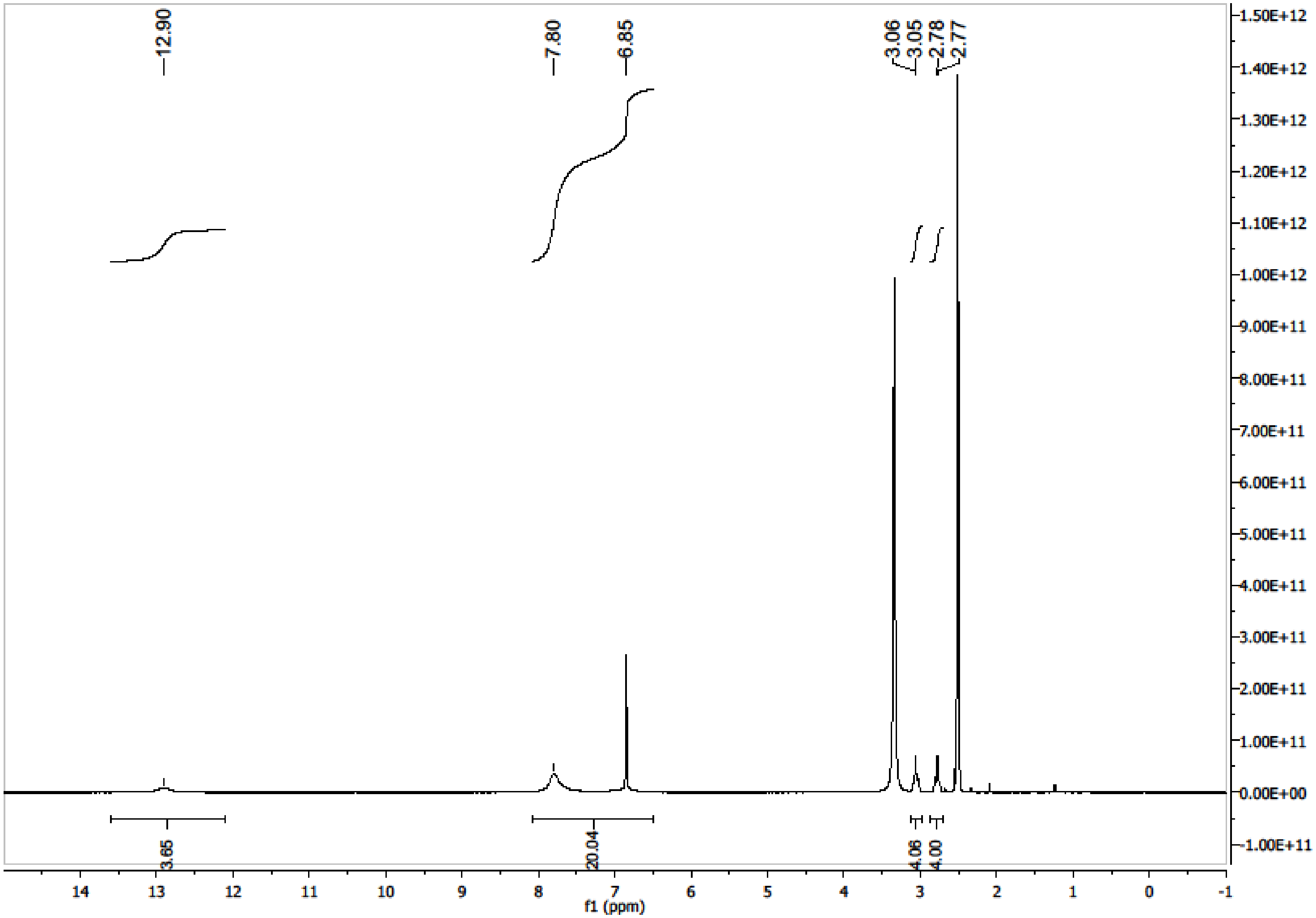

Figure S19– The ^{1}H NMR spectrum of **15**.

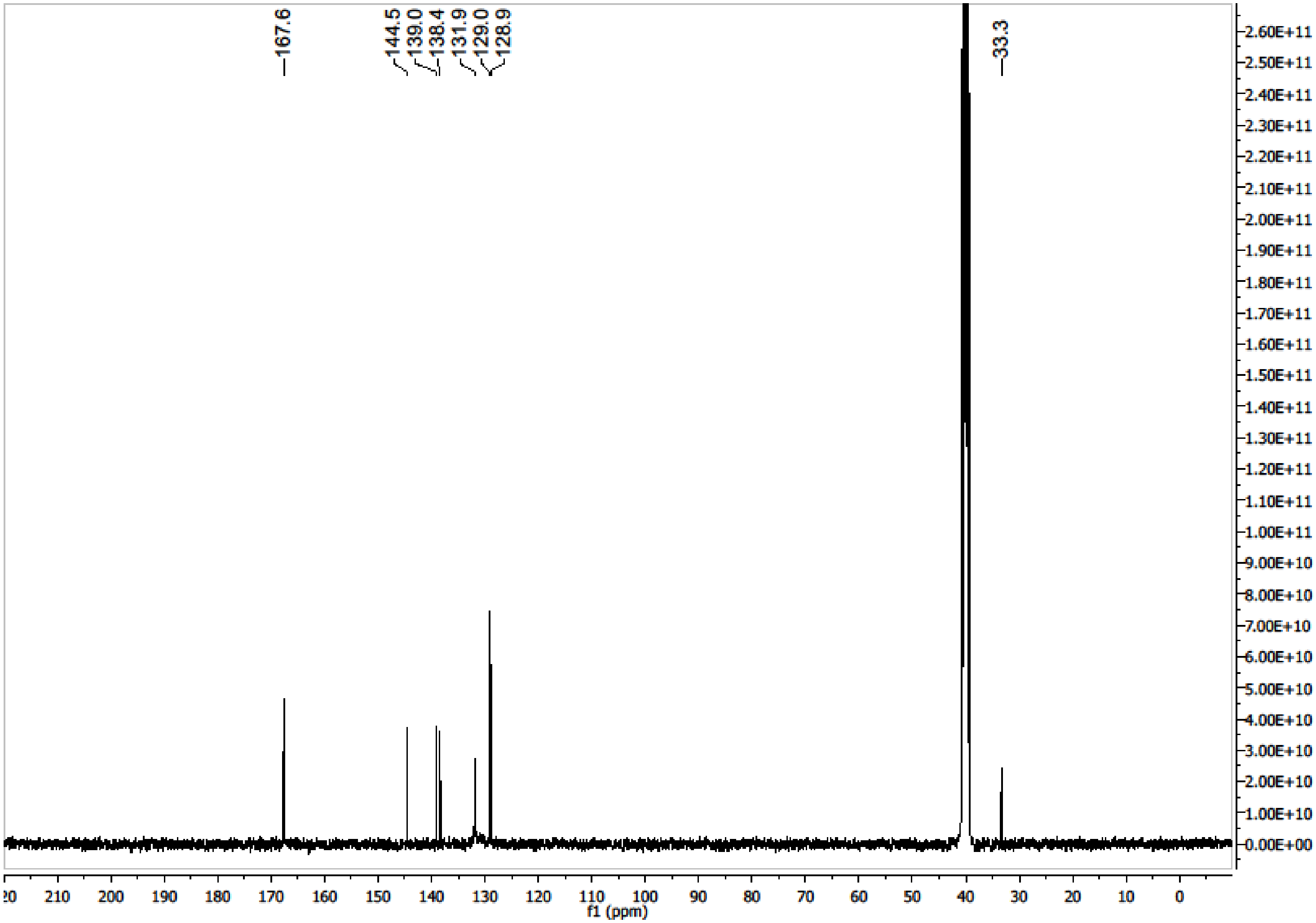

Figure S20– The ^{13}C NMR spectrum of **15**.

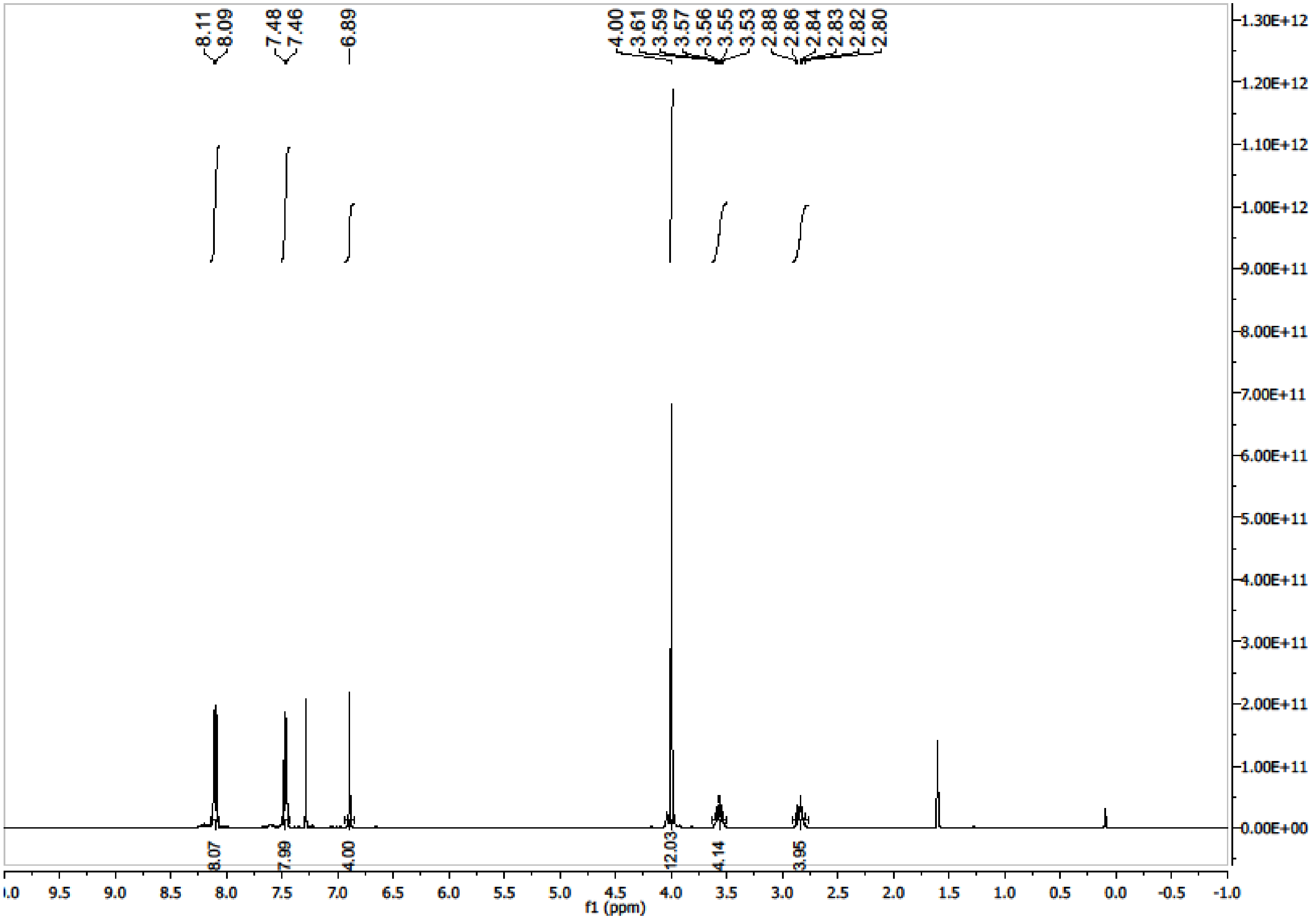

Figure S21– The ^{1}H NMR spectrum of **17**.

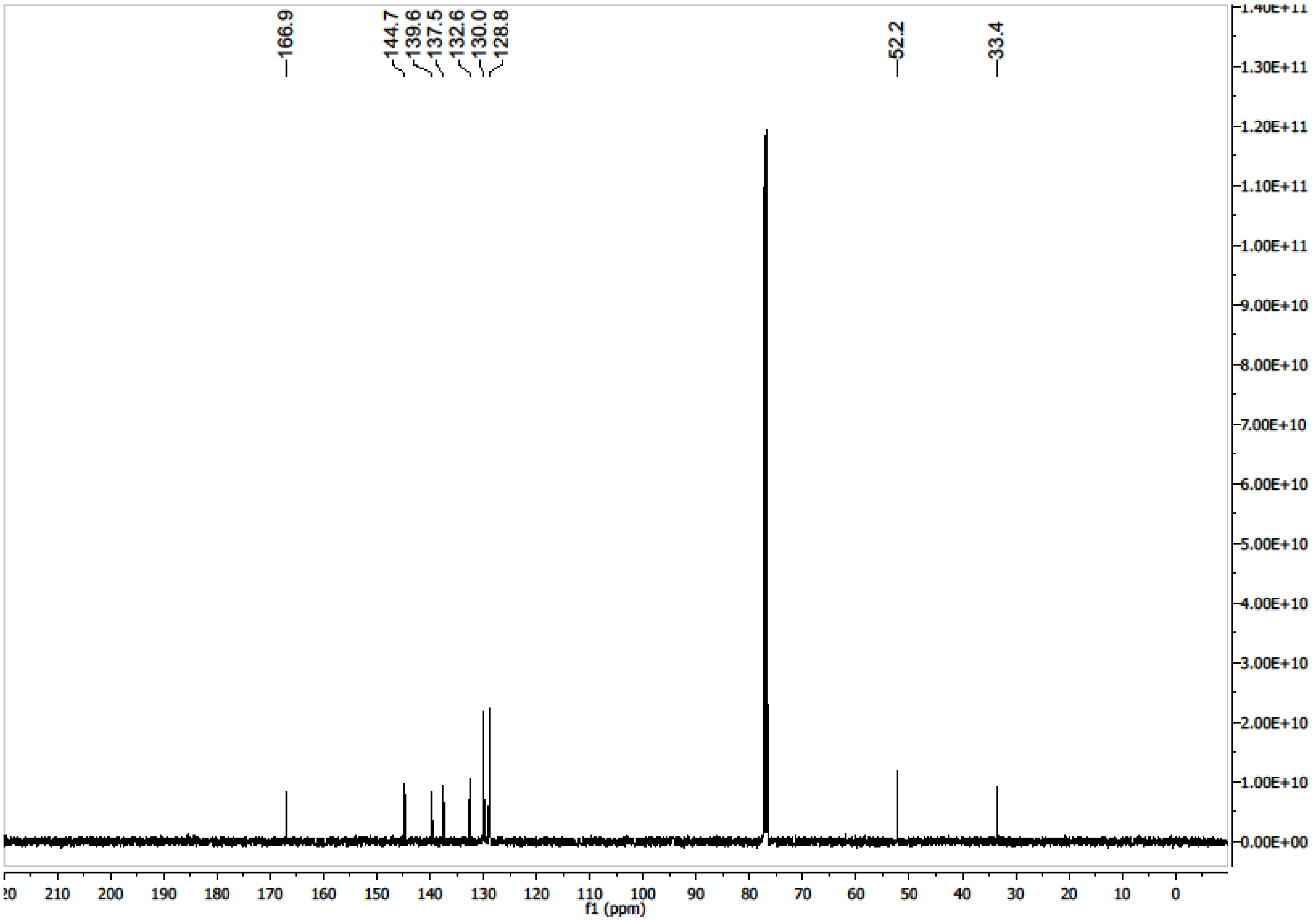

Figure S22– The ^{13}C NMR spectrum of **17**.

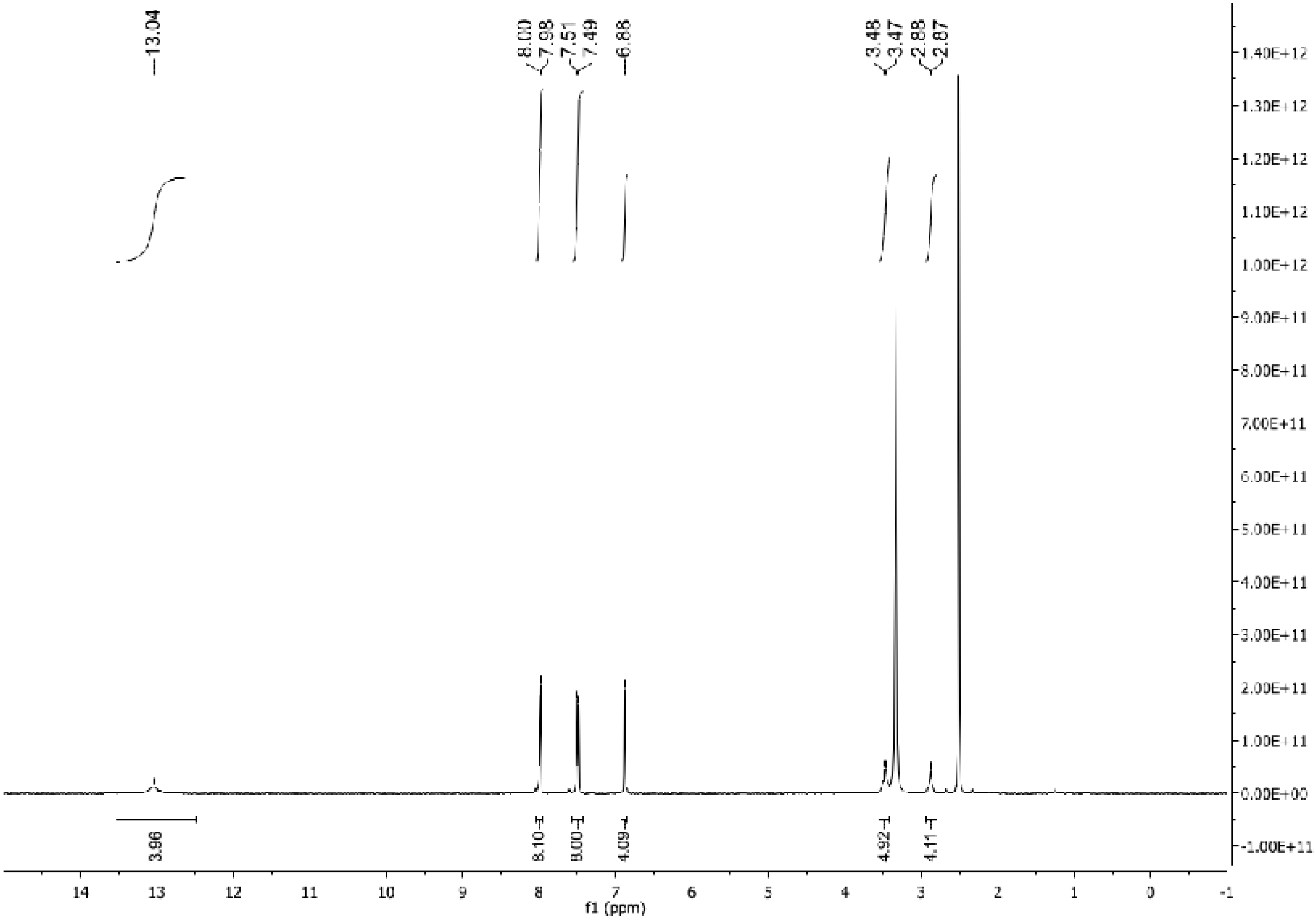

Figure S23– The ^{1}H NMR spectrum of **18**.

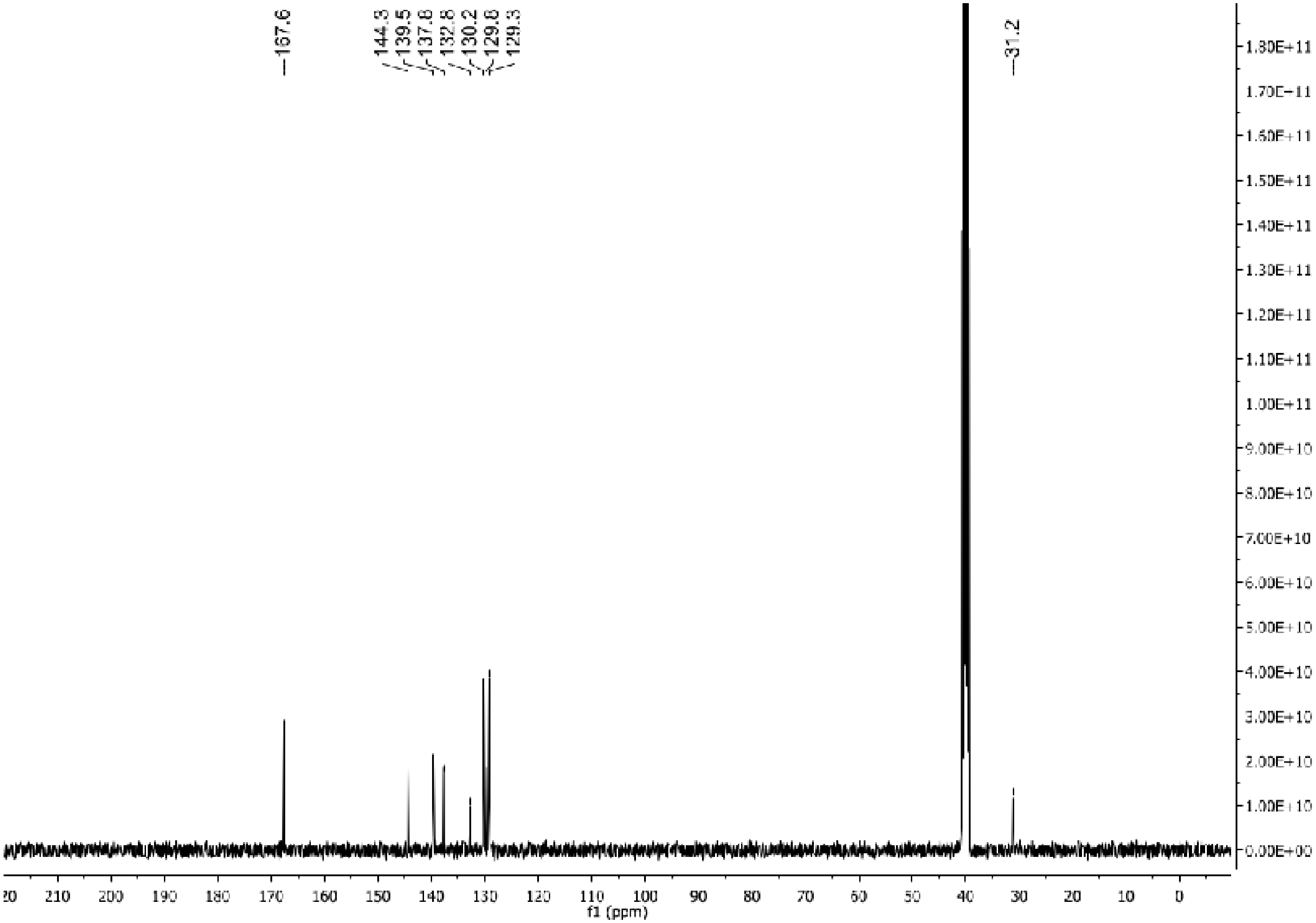

Figure S24– The ^{13}C NMR spectrum of **18**.

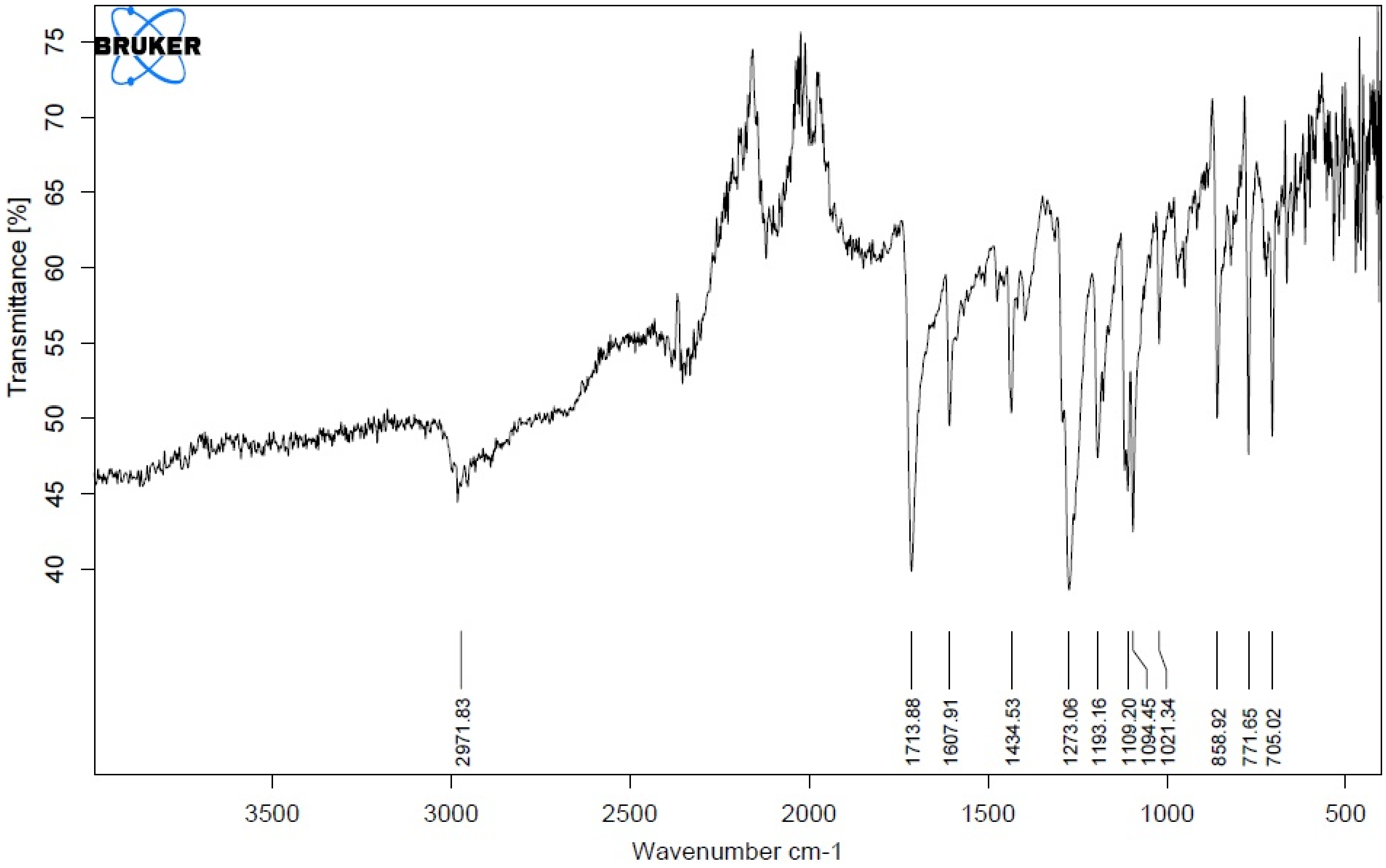

Figure S25 – The IR spectrum of **5**.

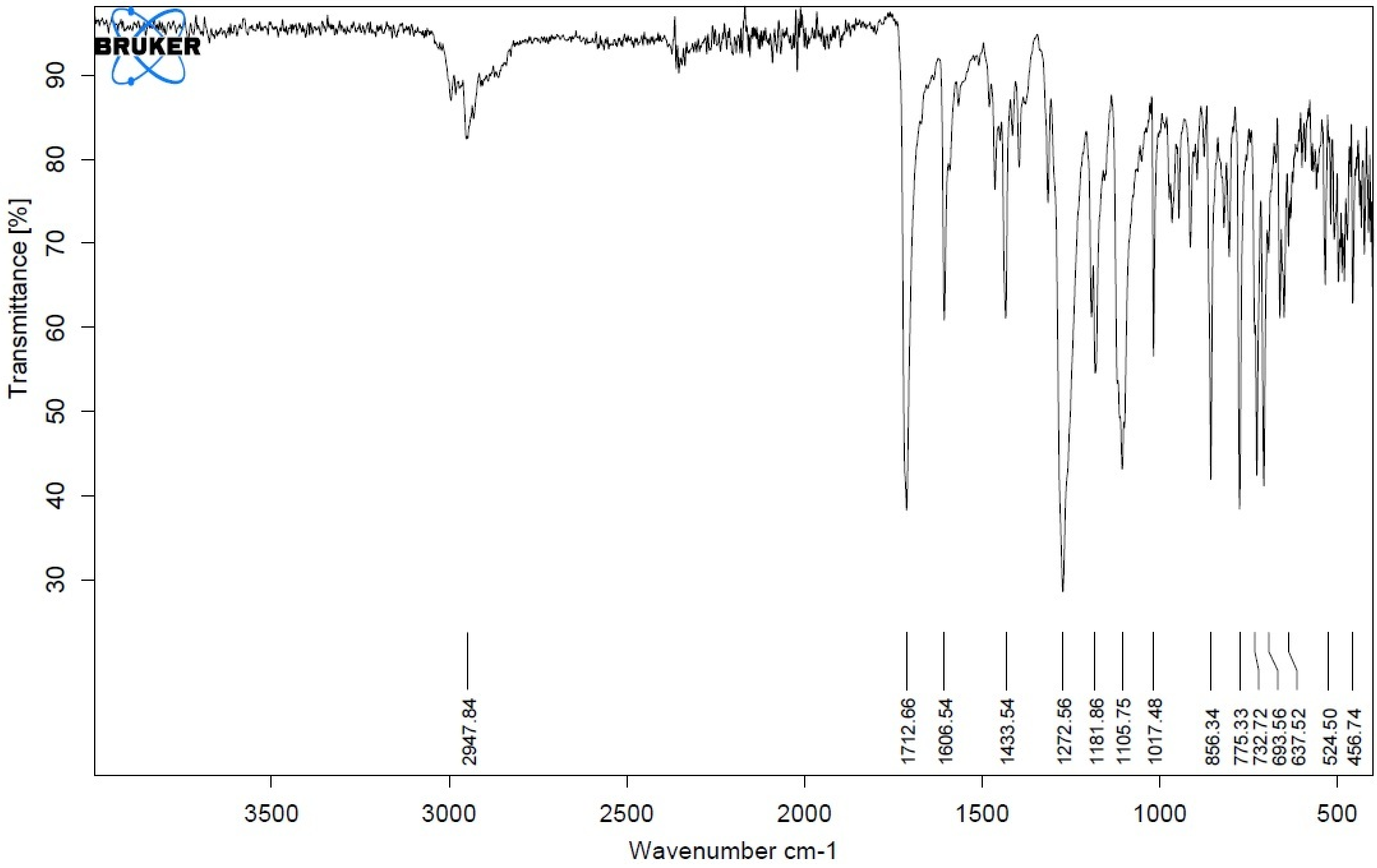

Figure S26 – The IR spectrum of **6**.

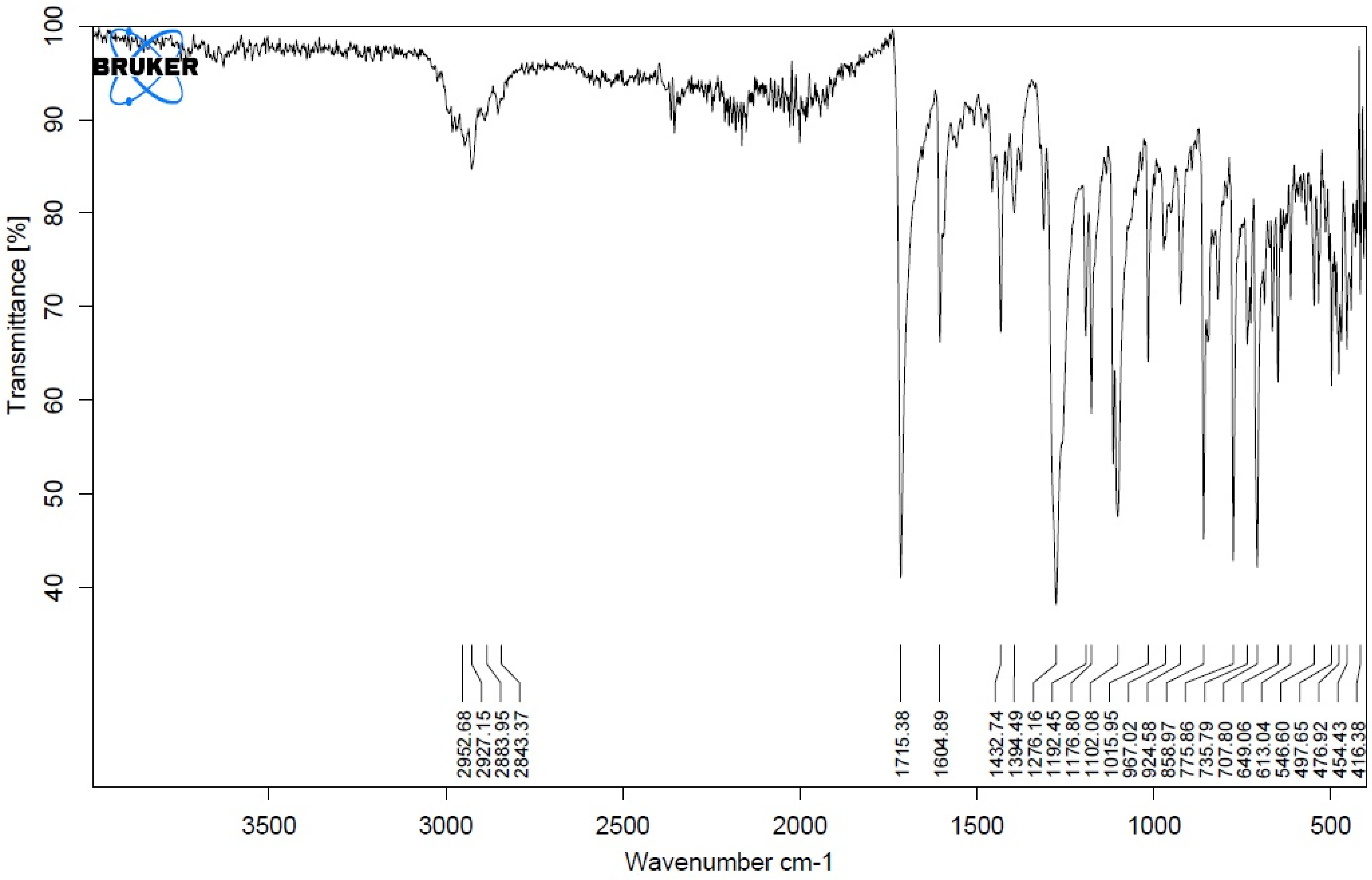

Figure S27 – The IR spectrum of **7**.

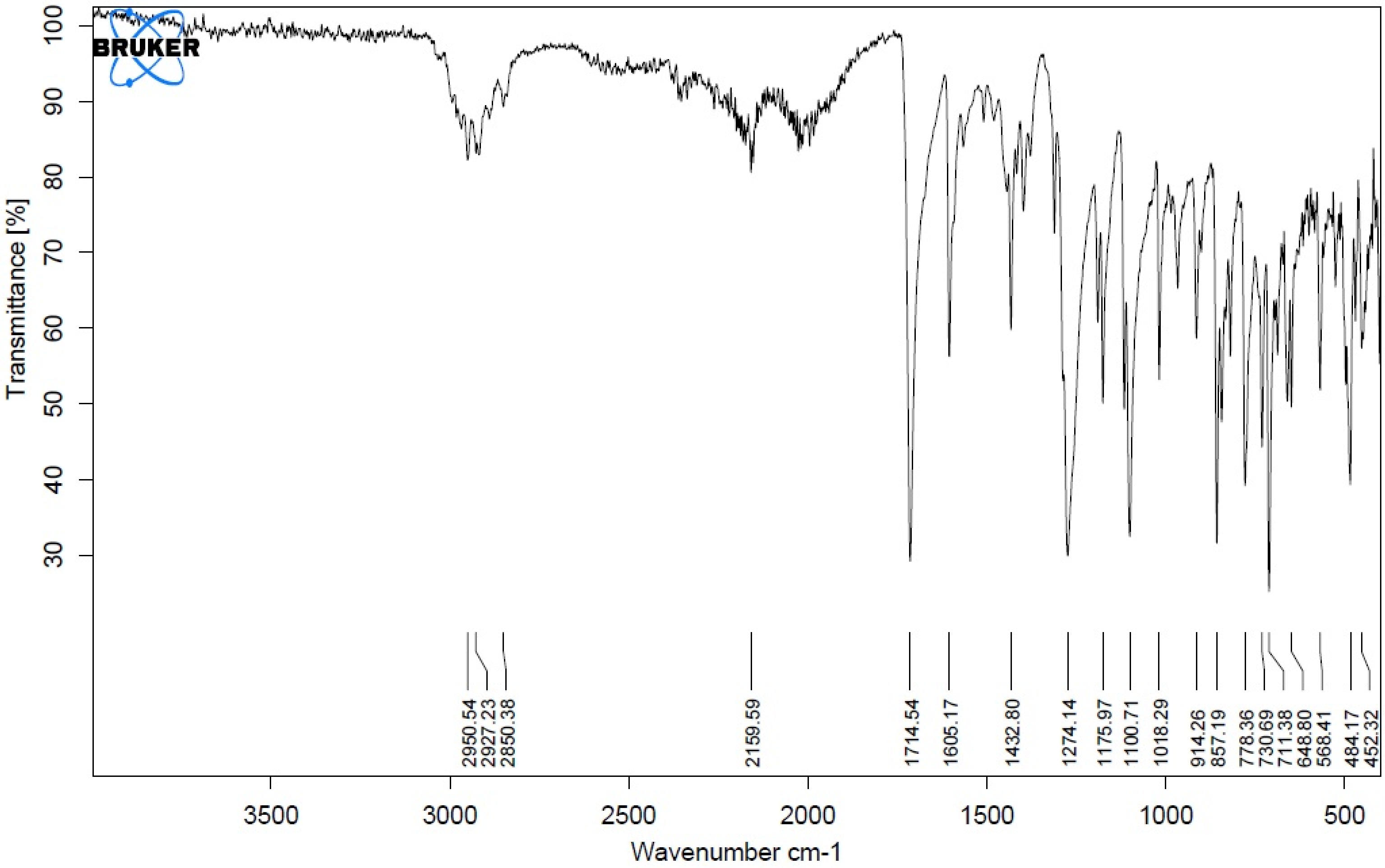

Figure S28 – The IR spectrum of **8**.

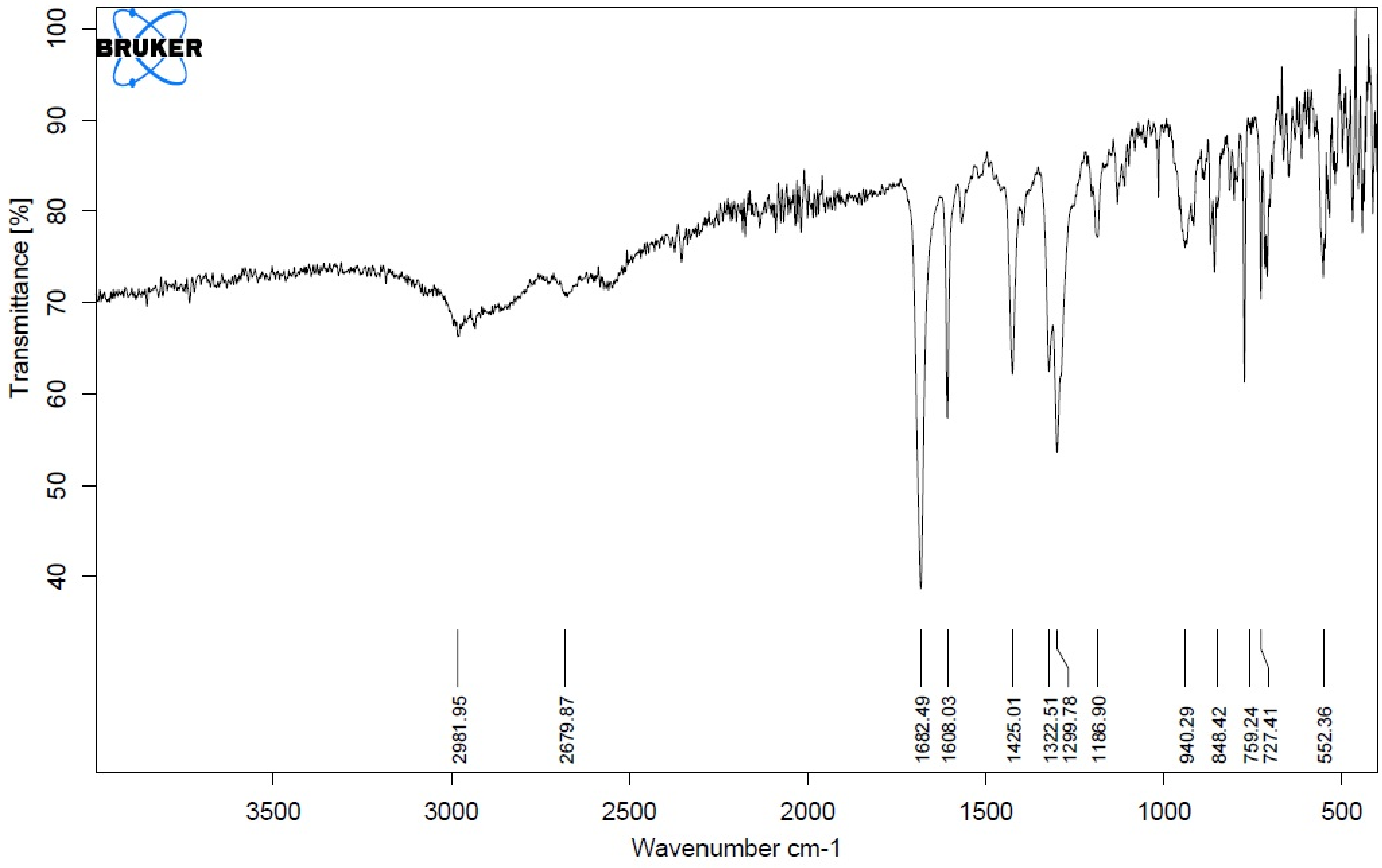

Figure S29 – The IR spectrum of **9**.

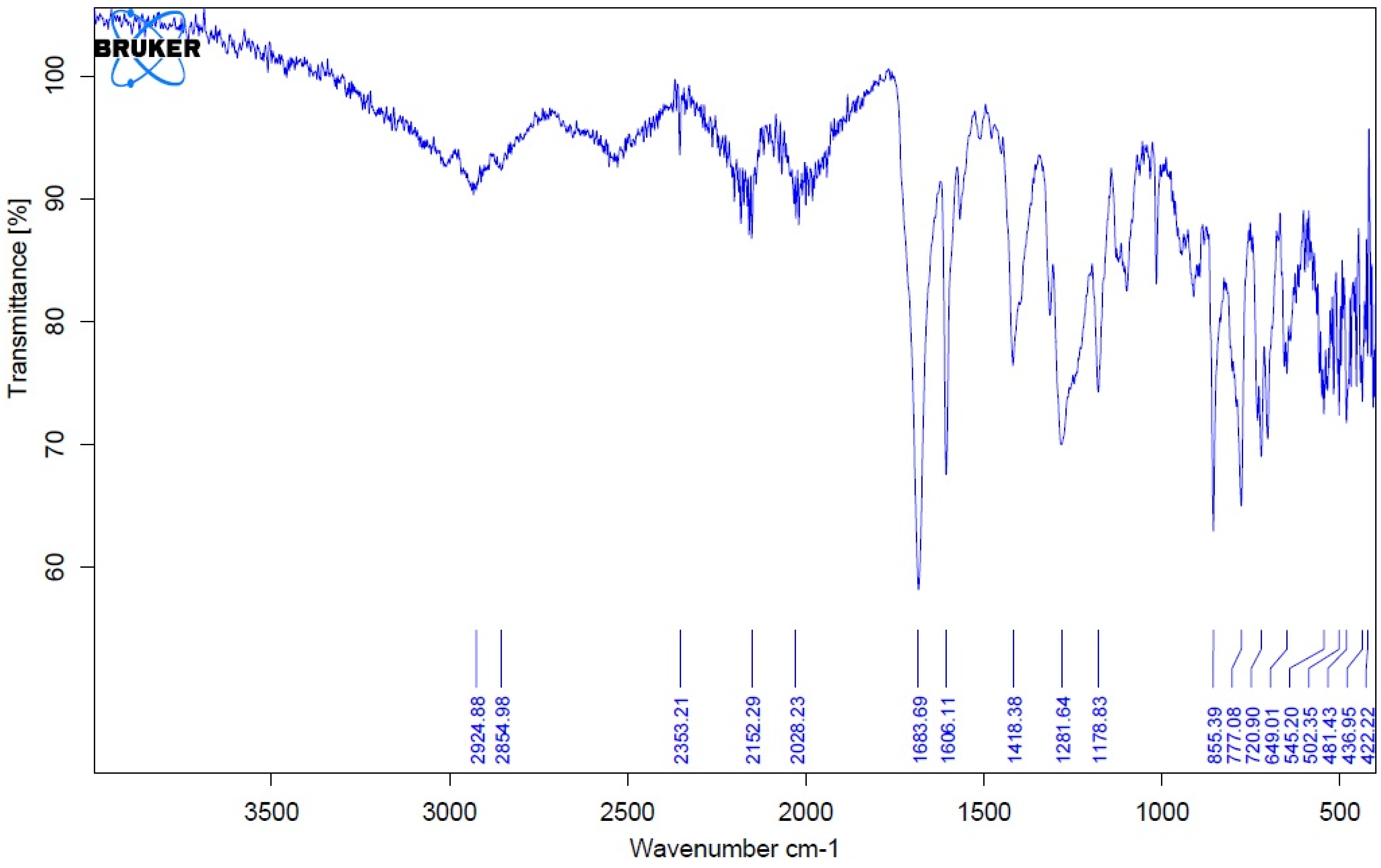

Figure S30 – The IR spectrum of **10**.

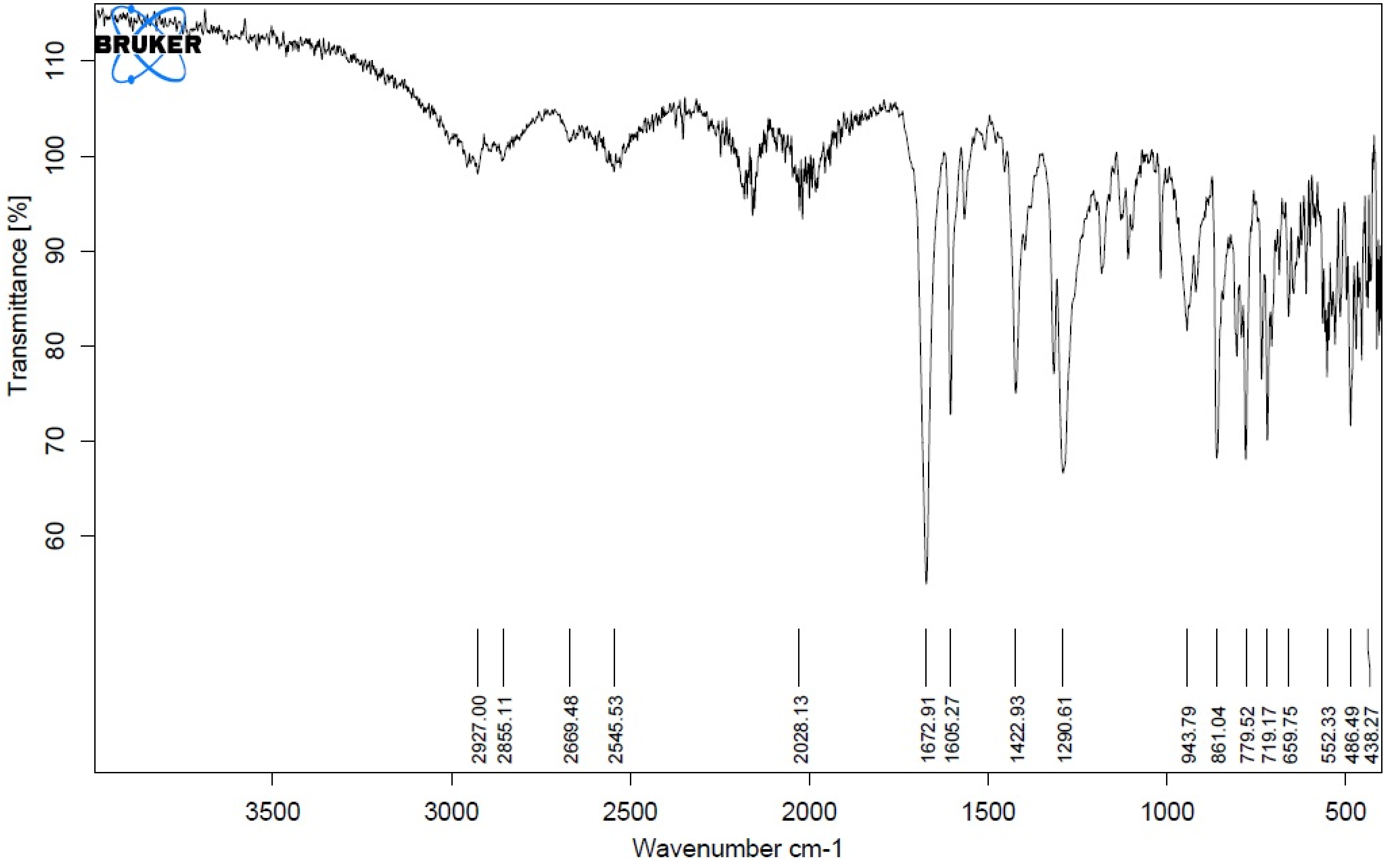

Figure S31 – The IR spectrum of **11**.

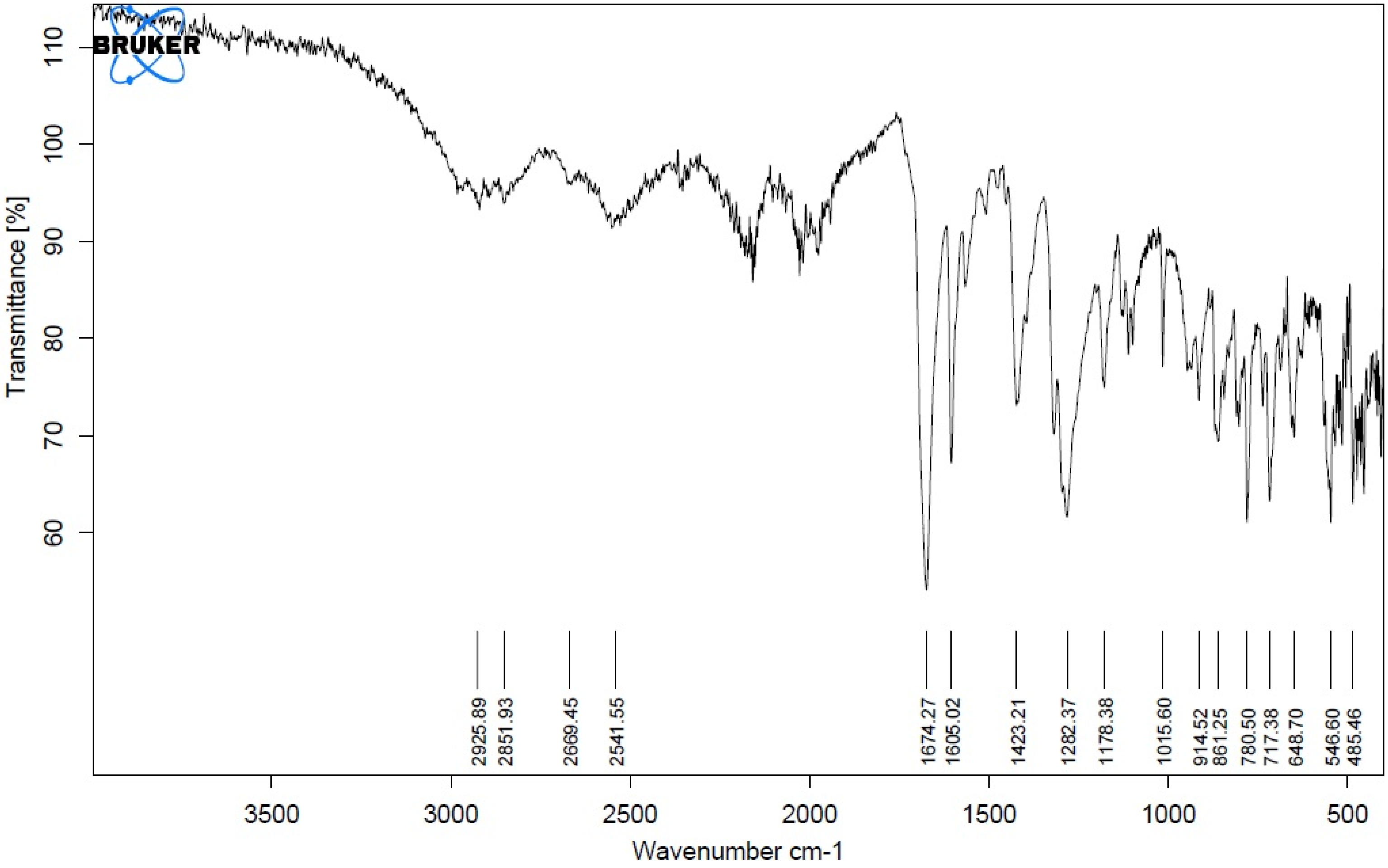

Figure S32 – The IR spectrum of **12**.

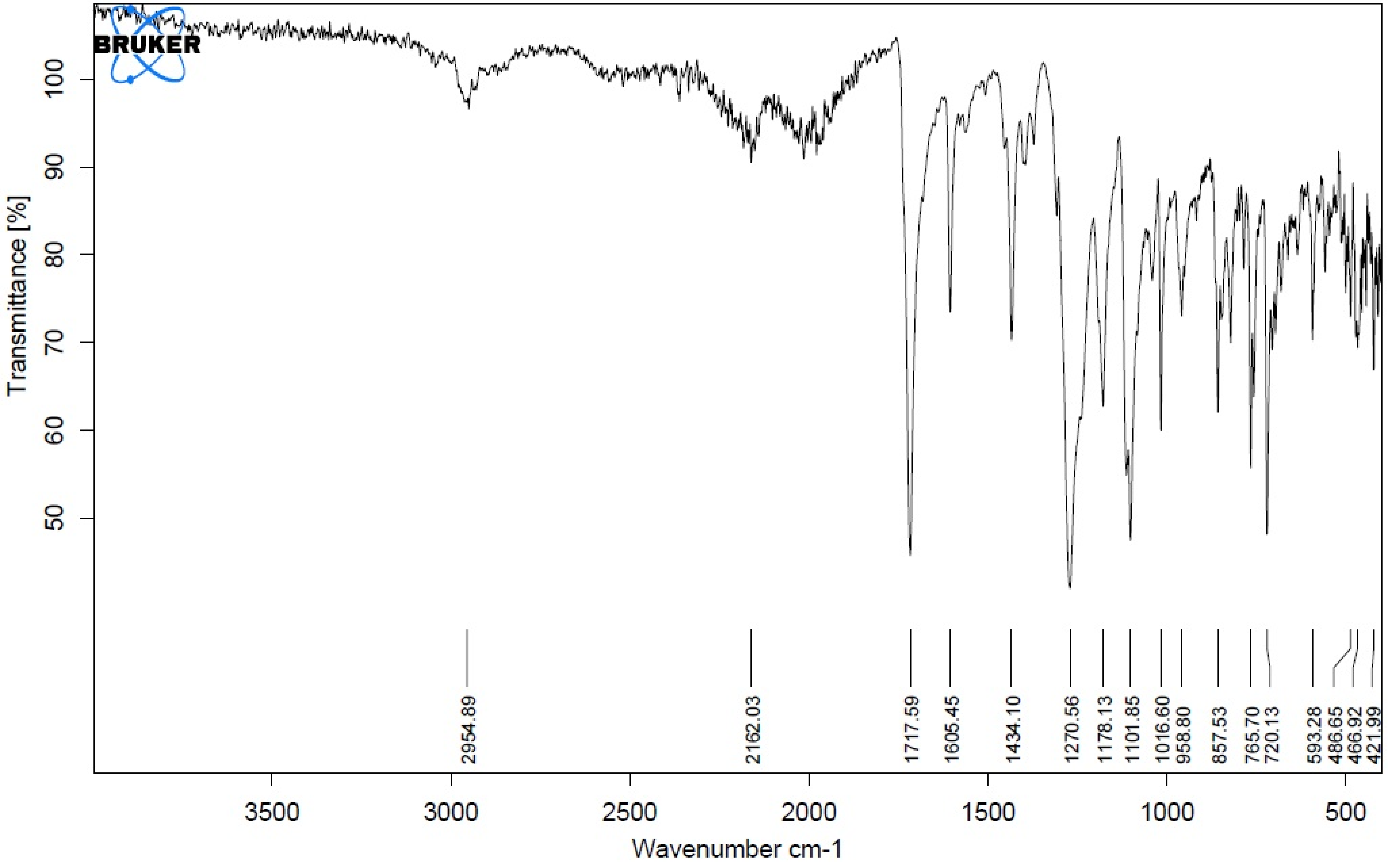

Figure S33 – The IR spectrum of **14**.

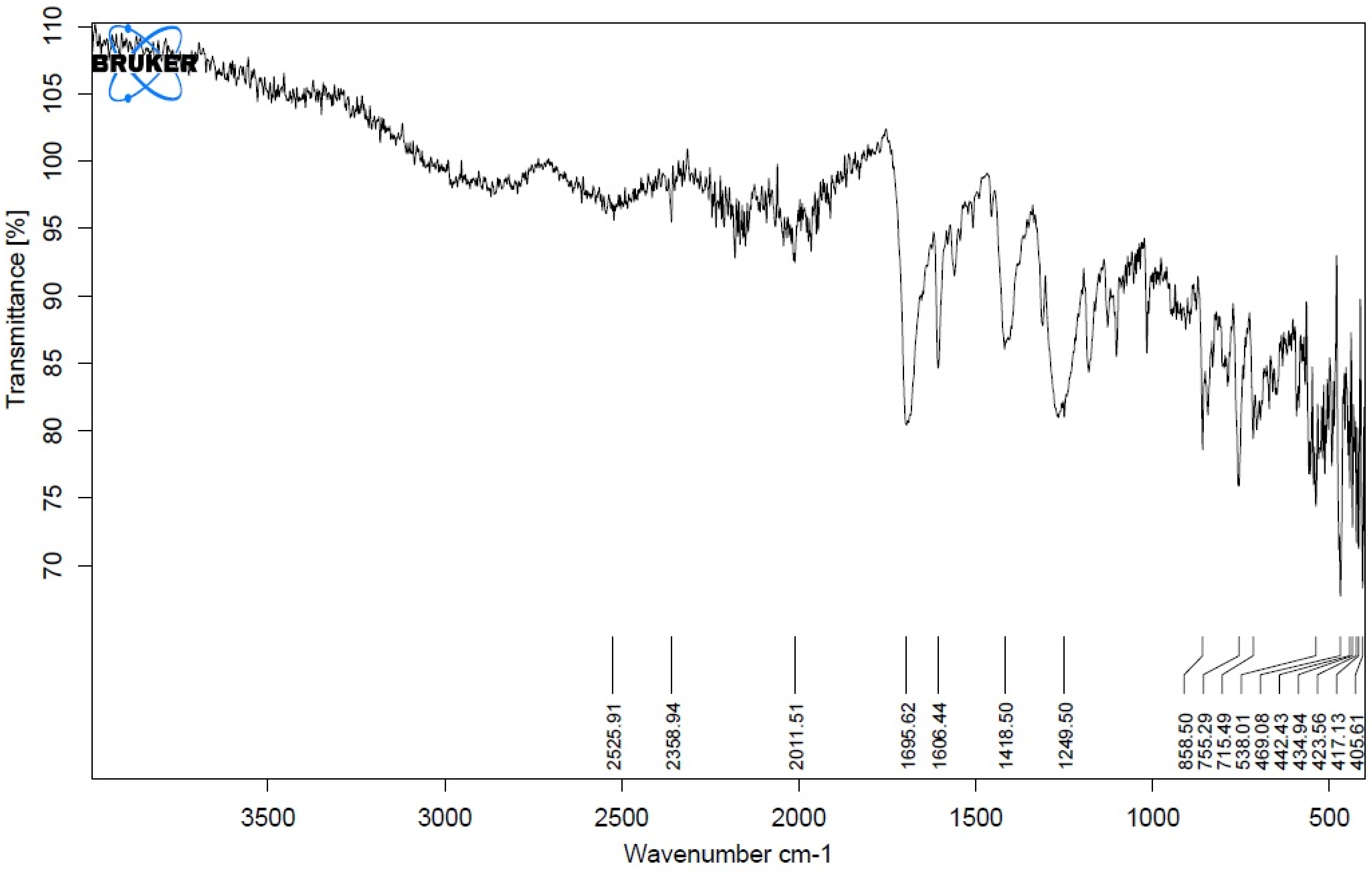

Figure S34 – The IR spectrum of **15**.

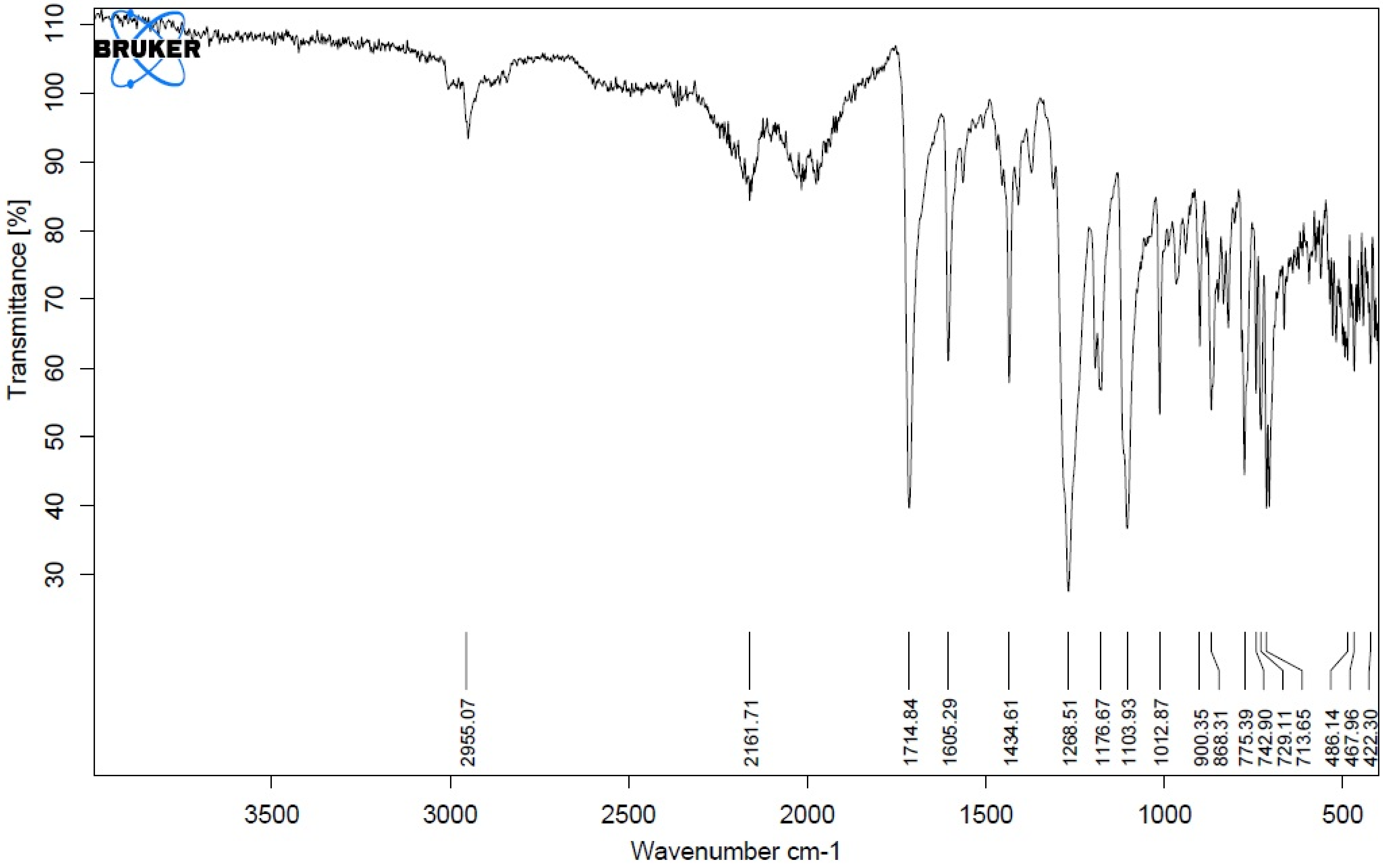

Figure S35 – The IR spectrum of **17**.

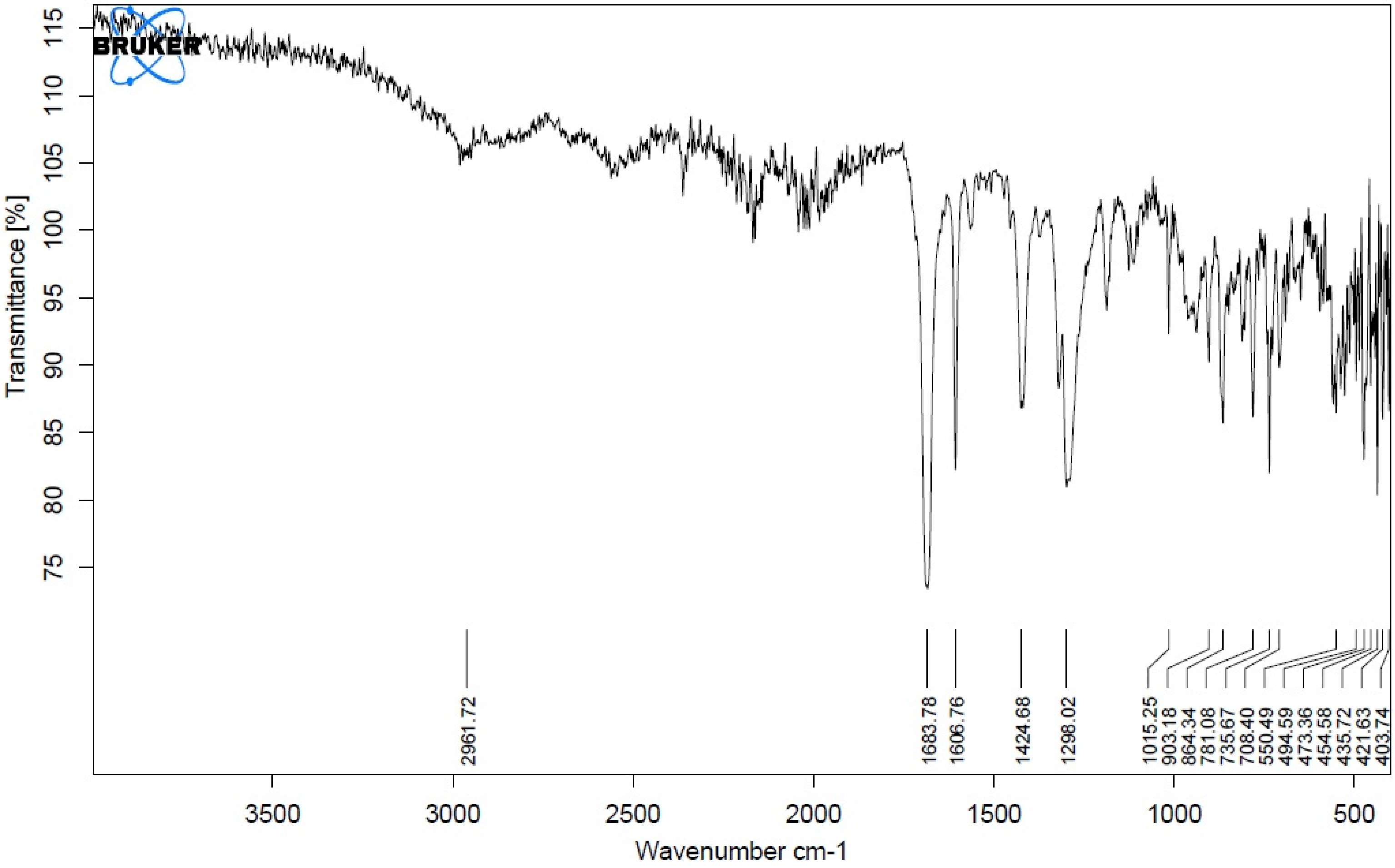

Figure S36 – The IR spectrum of **18**.

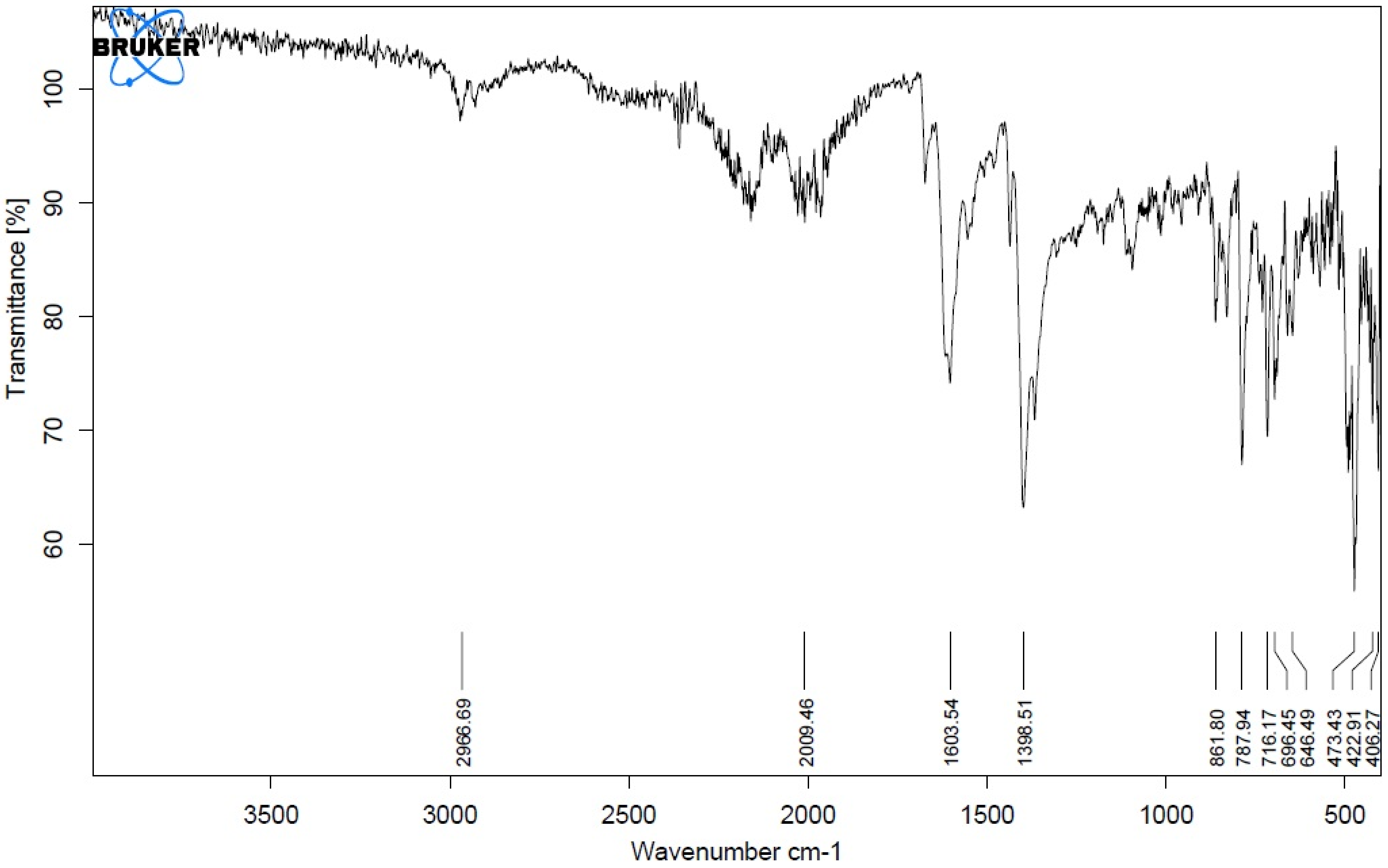

Figure S37 – The IR spectrum of **19**.
